# Crosstalk between CD8^+^ T cells and systemic bile acid metabolism shapes antiviral immunity and immunopathology

**DOI:** 10.1172/jci.insight.189882

**Published:** 2026-06-22

**Authors:** Felix Clemens Richter, Zsofia Keszei, Csilla Viczenczova, Maximilian Baumgartner, Henrique G. Colaço, Magdalena Siller, Lisa Holnsteiner, Hatoon Baazim, Anna Hofmann, Aubrey Burrett, Anna Schönbichler, Lukas Endler, Joel Xu En Wong, Laura Antonio-Herrera, Oleksandr Petrenko, Fabian Amman, Jakob-Wendelin Genger, Claudia D. Fuchs, Hubert Scharnagl, Hanns-Ulrich Marschall, Thomas Reiberger, Karl S. Lang, Clarissa Campbell, Michael Trauner, Andreas Bergthaler

**Affiliations:** 1 Department of Pathophysiology, Infectiology and Immunology, Medical University of Vienna, Vienna, Austria.; 2 CeMM Research Center for Molecular Medicine of the Austrian Academy of Sciences, Vienna, Austria.; 3 Institute of Immunology, University Hospital Essen, Essen, Germany.; 4 Sabri Ülker Center for Metabolic Research, Department of Molecular Metabolism, Harvard T.H. Chan School of Public Health, Boston, Massachusetts, USA.; 5 Animal Breeding and Genetics, University of Veterinary Medicine Vienna, Vienna, Austria.; 6 Division of Gastroenterology and Hepatology, Department of Medicine III, and; 7 Vienna Hepatic Experimental Hemodynamic (HEPEX) Laboratory, Division of Gastroenterology and Hepatology, Department of Medicine III, Medical University of Vienna, Vienna, Austria.; 8 Ukrainian Institute for Systems Biology and Medicine, Kyiv, Ukraine.; 9 Clinical Institute of Medical and Chemical Laboratory Diagnostics, Medical University of Graz, Graz, Austria.; 10 Wallenberg Laboratory and Department of Molecular and Clinical Medicine, Institute of Medicine, Sahlgrenska Academy, University of Gothenburg, Gothenburg, Sweden.

**Keywords:** Hepatology, Immunology, Metabolism, Hepatitis, T cells

## Abstract

Antiviral immunity profoundly impacts host metabolism, which can, in turn, modulate immune responses and influence disease pathology. The liver orchestrates systemic bile acid (BA) metabolism, a pathway disrupted in chronic liver diseases such as viral hepatitis. BAs are increasingly recognized for their immunomodulatory properties. Thus, improved understanding of the interplay between immunity and BA metabolism may reveal novel therapeutic avenues. Using lymphocytic choriomeningitis virus (LCMV) as a model, we investigated the interplay between chronic virus infection, BA metabolism, and immunity. Chronic LCMV infection increased BA levels and shifted circulating and liver BA composition toward host-derived, conjugated BAs. Hepatic BA transport and synthesis genes were broadly downregulated, in part depending on CD8^+^ T cells. Pharmacological inhibition of the main hepatic transporter of conjugated BAs, NTCP (*Slc10a1*), increased hepatic damage, while combined genetic disruption of the BA transporters *Slco1a1*, *Slco1a4*, and *Slco1b2*, responsible for the hepatic reuptake of unconjugated BA, reduced liver pathology and impaired antiviral CD8^+^ T cell responses. These findings reveal a reciprocal interplay between BA metabolism and CD8^+^ T cells, expanding our understanding of adaptive immunity in viral hepatitis. They also highlight how immunometabolic changes in liver disease may affect adaptive immune responses against infections.

## Abstract 2

Chronic viral hepatitis disrupts bile acid metabolism, which in turn affects CD8+ T cell responses, highlighting crosstalk between liver function, immunity, and disease outcomes.

## Graphical abstract

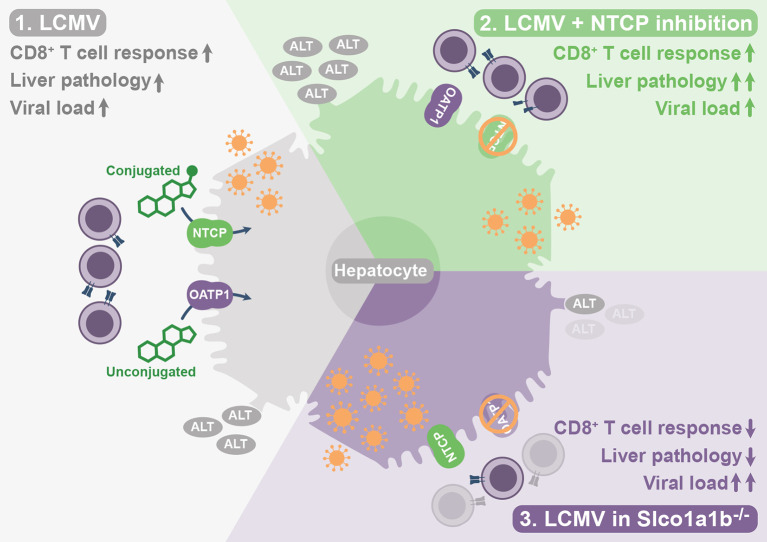

## Introduction

Liver disease causes about two million deaths each year — around 4% of all deaths globally — making it a leading cause of mortality. Viral hepatitis still accounts for most of these cases, followed by alcoholic liver disease and metabolic dysfunction–associated steatotic liver disease (formerly known as non-alcoholic fatty liver disease), both of which are expected to increase in numbers worldwide (1).

The liver is a critical metabolic hub that regulates systemic immunity by modulating the levels of local and circulating immunometabolites (2, 3), such as bile acid (BA) species (4). BAs are amphipathic cholesterol derivatives that circulate between the liver and the intestine. In hepatocytes, they are conjugated with taurine or glycine, reducing their toxicity and increasing their solubility, thereby promoting micelle formation and intestinal lipid absorption (4). Conjugated BAs are secreted via the bile salt export pump (BSEP), encoded by *Abcb11*, and released into the duodenum upon feeding. Host-derived (also known as primary) BAs can be modified by the gut microbiota into microbe-derived (also known as secondary) BAs, beginning with deconjugation by bacterial bile salt hydrolases (BSHs) (5). More than 95% of BAs are reabsorbed by ileal enterocytes and return to the liver via the portal vein blood (6). Hepatic BA reuptake is mediated by basolateral transporters, including the Na^+^-taurocholate cotransporting polypeptide (NTCP), encoded by *Slc10a1*, and the organic anion–transporting polypeptides (OATPs), specifically OATP1B2 (encoded by *Slco1b2*), OATP1A1 (encoded by *Slco1a1*), and OATP1A4 (encoded by *Slco1a4*) in mice (7). While there is considerable overlap in substrate specificity, both NTCP and OATPs play a critical role in the uptake of conjugated and unconjugated BAs, respectively (8–10). Genetic ablation of these transporters alters circulating BA composition, and their suppression by inflammation may protect hepatocytes from BA overload (7, 10–13).

De novo BA synthesis in hepatocytes is controlled by an enterohepatic feedback loop. In the ileum, BAs are taken up by the apical sodium-dependent bile acid transporter (ASBT; encoded by *Slc10a2*) and activate farnesoid X receptor (FXR; encoded by *Nr1h4*), inducing fibroblast growth factor 15 (*Fgf15*) expression. Secreted FGF15 signals to the liver via fibroblast growth factor receptor 4 (FGFR4) to suppress BA synthesis (14). FGFR4 activation promotes expression of the transcriptional corepressor small heterodimer partner (SHP; encoded by *Nr0b2*), leading to the repression of the rate-limiting BA synthesis enzyme *Cyp7a1*. Hepatic BA sensing by FXR can also directly induce *Nr0b2* expression (15, 16). Several BA transporters are regulated by FXR or SHP, contributing to hepatocellular homeostasis by limiting BA uptake (17–20).

Liver diseases, including viral hepatitis, are associated with disruptions in BA metabolism and altered BA composition (21–24). Changes in the BA pool can profoundly affect innate (25, 26) and adaptive immune cell functions. Microbe-derived BA species modulate CD4^+^ T cell subsets by promoting intestinal regulatory T cell formation and function (27–29). They also influence CD8^+^ T cells by regulating both their metabolism and effector function via FXR or TCR signaling (30–33). Given the central role of CD8^+^ T cells in antiviral immunity, understanding their regulation by BAs is critical. However, how BA composition changes during chronic viral infection and whether this shapes the antiviral immune response remain unknown.

To explore the link between liver diseases and BA dysregulation, we used the chronic viral hepatitis model lymphocytic choriomeningitis virus clone 13 (LCMV Cl13), which triggers a CD8^+^ T cell–mediated immunopathology (34–36). We examined how chronic infection alters BA metabolism and how disruption of the enterohepatic loop affects cytotoxic CD8^+^ T cell response and liver injury. LCMV infection increased systemic BA levels and shifted BA composition. In parallel, genes involved in hepatic BA metabolism, including those encoding the key reuptake transporter NTCP and OATPs, were substantially downregulated, and this regulation was partially dependent on CD8^+^ T cells. To assess the functional consequences, we used pharmacological and genetic models to disrupt hepatic BA reuptake. Pharmacological inhibition of the conjugated BA transporter NTCP elevated systemic BA levels and aggravated hepatic injury with minimal effects on the CD8^+^ T cell responses. In contrast, genetic deletion of the unconjugated BA transporter family OATP (encoded by *Slco1a1*, *Slco1a4*, and *Slco1b2*) impaired CD8^+^ T cell expansion and reduced liver immunopathology upon LCMV infection. Together, these findings demonstrate a reciprocal, context-dependent interplay between CD8^+^ T cells and systemic BA metabolism during viral hepatitis.

## Results

### Chronic LCMV infection shifts BA levels and composition.

**Figure 1:**
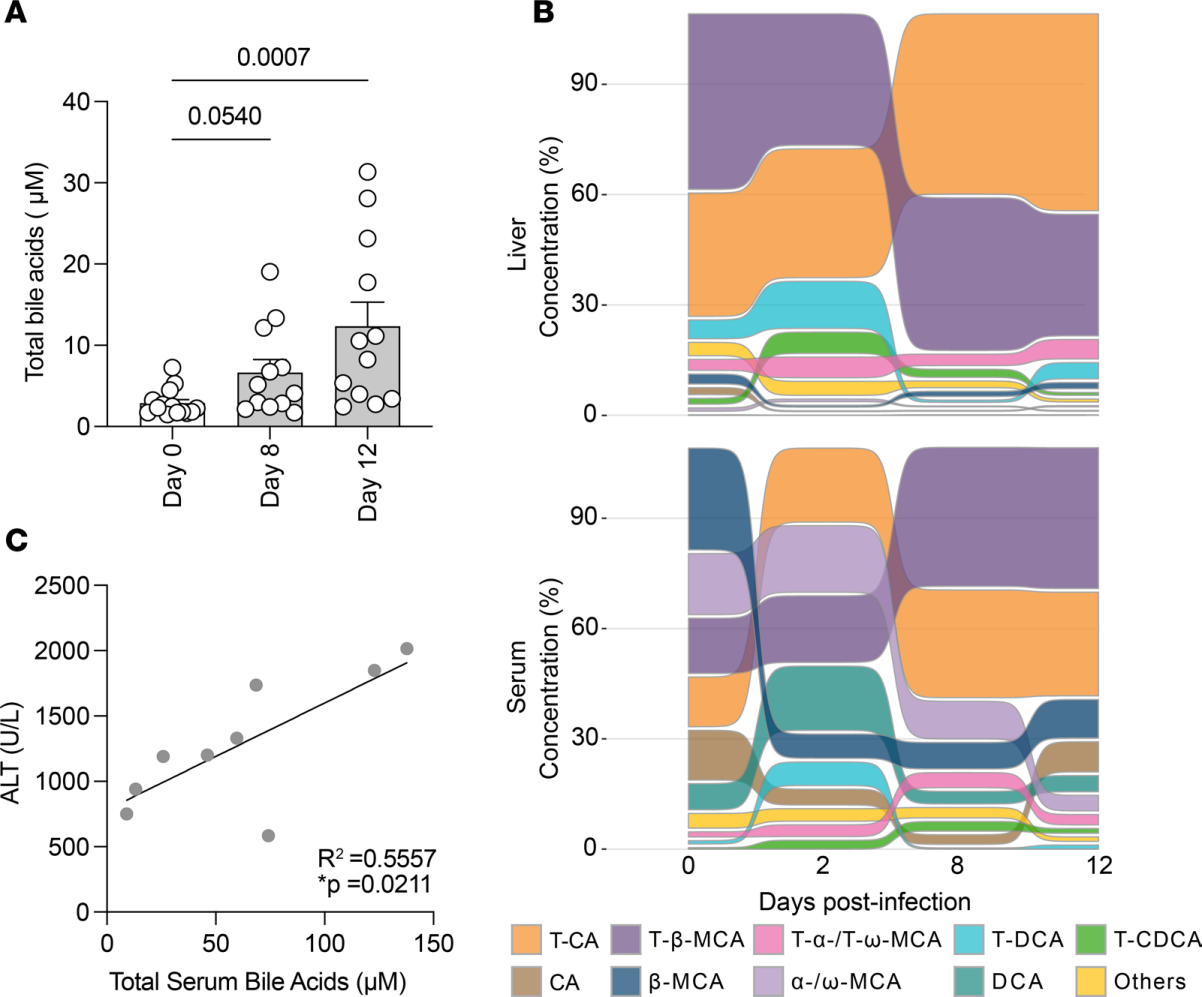
Viral hepatitis alters hepatic and systemic BA levels and composition. (**A**) Total serum BA levels of C57BL/6J mice infected with LCMV Cl13 quantified by colorimetric total BA assay. Data were pooled from 4 independent experiments (*n* = 12–14 mice per group) and analyzed with Kruskal-Wallis test. (**B**) Composition of serum and liver BA species in uninfected and LCMV Cl13–infected mice at 8 and 12 days after infection measured by LC-MS. The bump plot shows the relative proportion of each BA and its rank at distinct time points including at day 0 (uninfected), day 2, day 8, and day 12. Data are representative of 2 independent experiments (*n* = 5 mice per group). (**C**) Correlation of the hepatic damage marker ALT with systemic BA levels in the serum of C57BL/6J mice infected with LCMV Cl13 at day 12 after infection measured by LC-MS. Data were pooled from 2 independent experiments (*n* = 9 mice) and analyzed as simple linear regression.

To establish whether BA metabolism is affected by viral hepatitis, we quantified total BA species in the serum of mice infected with LCMV Cl13. We observed an increase in total serum BAs, which was most pronounced 12 days after infection (Figure 1A), confirming previous observations with the LCMV strain WE (37). To validate our results and gain new insights into the composition of BA levels during LCMV infection, we performed a more sensitive analysis by targeted liquid chromatography/mass spectrometry (LC-MS) across several time points on liver and serum samples. Supporting our initial findings, we observed a profound alteration in BA profiles as the viral infection progressed (Figure 1B). This shift was marked by an enrichment of predominantly host-derived and conjugated BAs in both serum and liver, which was most pronounced 8 days after infection (Supplemental Figure 1, A and B; supplemental material available online with this article; https://doi.org/10.1172/jci.insight.189882DS1), which coincides with the peak of CD8^+^ T cell responses (38).

Host-derived conjugated BAs are deconjugated by BSHs in the lower gastrointestinal tract. This process is pivotal for subsequent microbial modifications (5, 39). During LCMV infection, the gut microbiota undergoes a substantial compositional shift, causing gut dysbiosis (40). To examine whether the shift toward host-derived conjugated BA species may be caused by a decrease of BSH-expressing gut microbes, we performed 16S rRNA sequencing on mice infected with LCMV Cl13 or uninfected control mice and isolated fecal and cecal samples at day 8 after infection. Microbial composition was subsequently analyzed using the PICRUSt algorithm (41). Interestingly, the relative abundance of BSH-expressing bacteria was unchanged in both fecal and cecal samples (Supplemental Figure 1, C and D). In line with these findings, measurement of ex vivo BSH activity was comparable between uninfected and LCMV Cl13–infected mice (Supplemental Figure 1E). Collectively, this suggests that the accumulation of host-derived conjugated BAs in the serum and liver is likely not driven by an inability of the microbiota to process these BAs.

Alternatively, it seemed likely that the systemic accumulation of BAs could result from a direct effect of liver damage (12). Indeed, there was a positive correlation between the hepatocyte damage marker alanine aminotransferase (ALT) and the total concentration of BAs at day 12 after LCMV infection as quantified by LC-MS BA profiling (Figure 1C). Thus, direct release of host-derived conjugated BAs into the bloodstream due to hepatocyte damage may contribute to the observed shift in circulating BA profiles.

### Chronic LCMV infection decreases hepatic BA metabolism gene expression.

**Figure 2:**
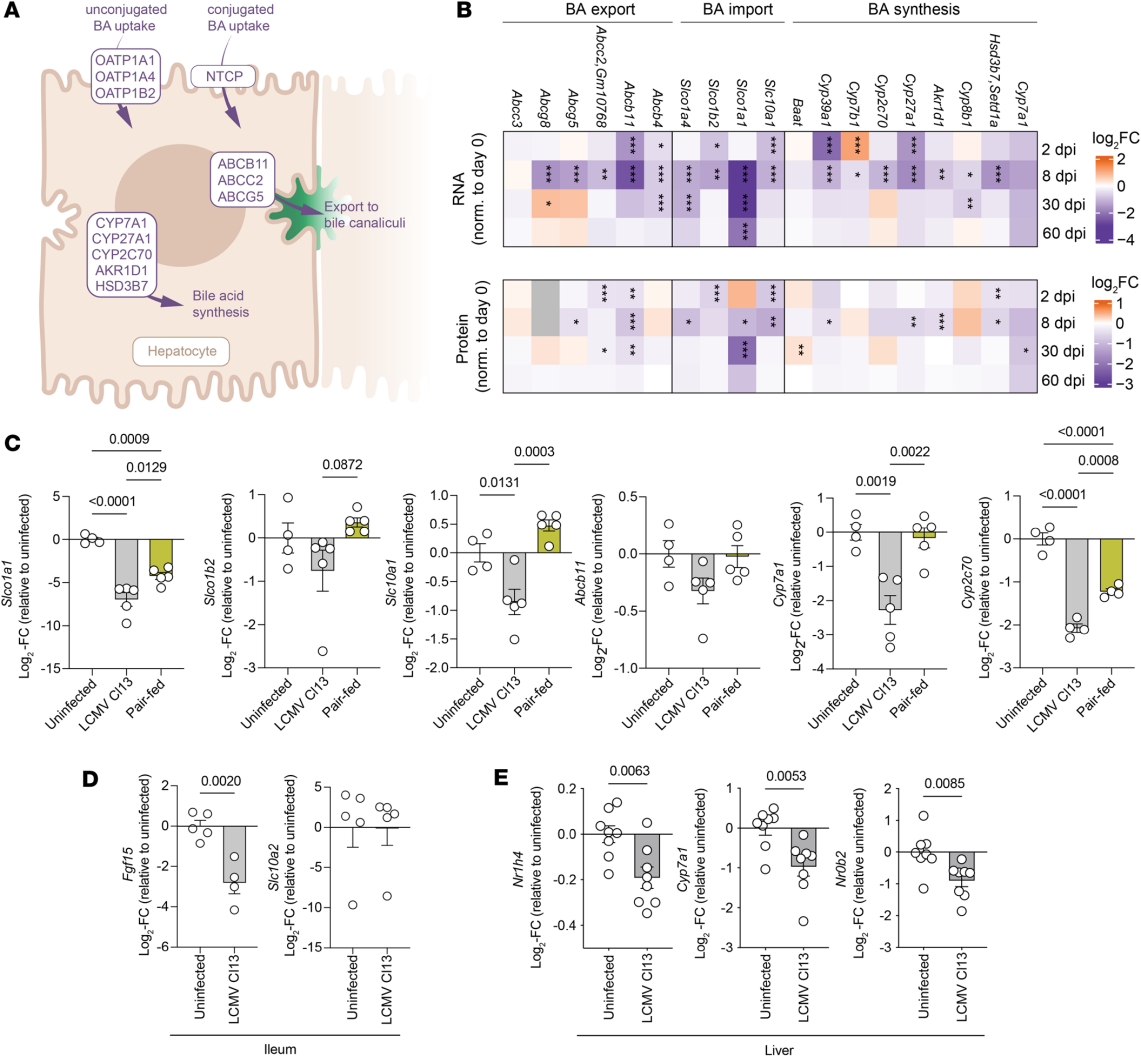
Chronic LCMV infection decreases the expression of genes involved in hepatic BA regulation, synthesis, and transport. (**A**) Overview schematic of BA homeostasis–related genes in hepatocytes that are significantly downregulated at both the transcript and protein levels at one or more time points. (**B**) Expression of BA homeostasis–related genes and proteins during infection with LCMV Cl13 infection based on Lercher et al. (42). FC, fold change. (**C**) Hepatic gene expression of BA-metabolic genes in response to LCMV Cl13 infection or pair feeding. Data are representative of 3 independent experiments (*n* = 4–5 mice per group) and were analyzed using 1-way ANOVA with post hoc Tukey’s multiple-comparison test. (**D**) Gene expression in ileum of uninfected mice and LCMV Cl13–infected mice at day 8 after infection. Data are representative of 3 independent experiments (*n* = 4–5 mice per group) and were analyzed using unpaired 2-tailed Student’s *t* test. (**E**) Expression of hepatic genes involved in FGF15/SHP signaling. Data were pooled from 2 independent experiments (*n* = 8 mice per group) and analyzed using unpaired 2-tailed Student’s *t* test.

In addition to the systemic changes in BA levels and composition, we wanted to assess the impact of LCMV infection on organismal BA metabolism. Using a published dataset (42), we investigated changes in hepatic BA metabolism by LCMV Cl13 infection at both the transcriptomic and the proteomic level. Kyoto Encyclopedia of Genes and Genomes (KEGG) pathway enrichment analysis revealed a broad metabolic reprogramming in the liver during viral infection, with numerous metabolism-related KEGG pathways downregulated, especially on day 8 after infection (Supplemental Figure 2A). In contrast, ample immune-related pathways were upregulated as expected (Supplemental Figure 2B). Bile secretion was among the most downregulated KEGG pathways, together with other terms related to fatty acid and steroid metabolism (Supplemental Figure 2A). Regulated genes in this pathway included those responsible for primary BA synthesis and basolateral BA transport from the blood to the hepatocyte, as well as bile secretion, as shown in the schematic (Figure 2A). Transcripts of basolateral BA uptake transporter genes (*Slc10a1*, *Slco1a1*, *Slco1a4*, *Slco1b2*), the canalicular BA export pump gene *Abcb11*, and BA biosynthesis genes (*Cyp7a1*, *Cyp27a1*, *Cyp2c70*, *Cyp39a1*) were all found to be downregulated at least at one time point in transcript and protein levels (Figure 2B and Supplemental Table 2).

We next sought to identify the driving factors behind these transcriptional changes. Chronic LCMV infection induces anorectic behavior (40, 43), which can substantially impact the regulation of BA metabolism and associated gene expression (14, 44). To confirm that the alterations in gene expression are not solely due to reduced food intake, we evaluated the expression of BA transporters and the rate-limiting BA synthesis enzyme *Cyp7a1* in uninfected mice that were pair-fed an amount of food equivalent to that consumed by their Cl13-infected counterparts. We observed similar expression levels in the liver of both uninfected and pair-fed mice (Figure 2C), except for *Slco1a1* and *Cyp2c70*, indicating that the alterations in transcript levels are not solely influenced by reduced food intake, but likely require an inflammatory response.

The downregulation of hepatic BA metabolism during chronic LCMV infection could result from an increased ileal FGF15 signaling via the hepatic FGFR4/SHP axis (14). Alternatively, hepatic BA sensing via FXR may directly induce *Nr0b2* (encoding SHP) or regulate BA synthesis and transport genes (15–20, 45). To assess the role of the FGF15/FGFR4/SHP axis, we measured target gene expression in ileum and liver. Ileal *Fgf15* expression was reduced, while the BA uptake transporter *Slc10a2*, encoding ASBT, remained unchanged (Figure 2D), arguing against increased FGF15 signaling. Consistent with this, hepatic *Nr1h4* (encoding FXR) and *Nr0b2* (encoding SHP) transcript levels were decreased (Figure 2E), as was the FXR-target gene *Cyp7a1* in LCMV Cl13–infected mice (Figure 2E). Together, these findings suggest that suppression of genes related to hepatic BA metabolism may be largely independent of ileal FGF15 signaling and reduced food intake.

### CD8^+^ T cells play an important role in downregulating BA transporter expression.

Infection with LCMV is characterized by an early type I interferon (IFN-I) response, a potent innate antiviral cytokine, which can drive substantial metabolic alterations in the liver (42). To test whether IFN-I signaling controls BA transporter expression during viral infection, we genetically ablated the IFN-α receptor (*Ifnar*) in the whole mouse (*Ifnar1^–/–^*) or specifically in hepatocytes (*Alb-Cre Ifnar1^fl/fl^*). Upon infection with LCMV Cl13, mice with either whole-body or hepatocyte-specific *Ifnar* deficiency exhibited similar reductions in hepatic BA transporter expression (Supplemental Figure 3, A and B) compared with littermate controls. This suggests that the downregulation of BA transporters is independent of IFN-I signaling.

**Figure 3:**
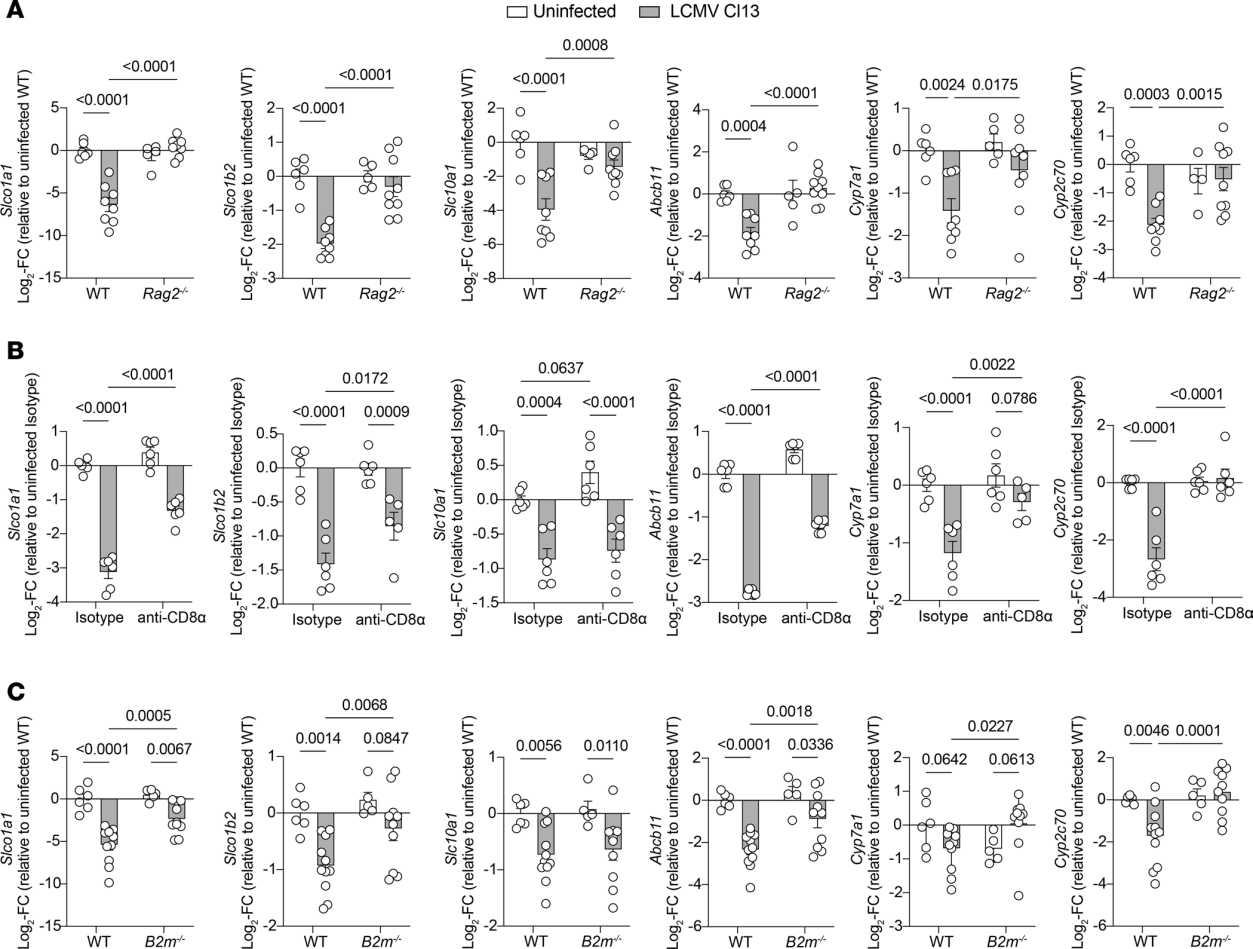
Downregulation of hepatic BA transporters is partially due to CD8^+^ T cells during LCMV Cl13 infection. (**A**) Hepatic expression of BA metabolism genes in response to LCMV Cl13 infection in C57BL/6J and *Rag2*-deficient mice at day 8 after infection. Data were pooled from 2 independent experiments (*n* = 5–9 mice per group) and analyzed using 2-way ANOVA with post hoc uncorrected Fisher’s least significant difference (LSD). (**B**) Hepatic BA metabolism gene expression in response to LCMV Cl13 infection and CD8α-depleting antibody administration at day 8 after infection. Data were pooled from 2 independent experiments (*n* = 6 mice per group) and analyzed using 2-way ANOVA with post hoc uncorrected Fisher’s LSD. (**C**) Hepatic BA metabolism gene expression in C57BL/6J and *B2m*-deficient mice in response to LCMV Cl13 infection at day 8 after infection. Data were pooled from 3 independent experiments (*n* = 5–11 mice per group) and analyzed using 2-way ANOVA with post hoc uncorrected Fisher’s LSD.

To address whether the adaptive immune response to Cl13 is required for the changes in BA-metabolic gene expression, we infected *Rag2^–/–^* mice, which are unable to produce mature T and B lymphocytes (46), with LCMV Cl13. Interestingly, *Rag2^–/–^* mice were protected from infection-associated downregulation of all tested genes (Figure 3A). This implicated the adaptive immune response in regulating genes involved in BA transport and synthesis. To further pinpoint the key players involved, we sought to determine whether CD8^+^ T cells drive the downregulation of these transporters. Administration of CD8α-depleting antibodies or genetic deletion of β_2_-microglobulin (*B2m*) successfully depleted CD8^+^ T cells from circulation (Supplemental Figure 3, C and D) and resulted in modulation of BA-metabolic gene expression similar to that seen in *Rag2^–/–^* mice, albeit with slightly attenuated outcomes (Figure 3, B and C).

CD8^+^ T cells can influence target cells through the secretion of various inflammatory cytokines. To assess the impact of CD8^+^ T cell effector cytokines on BA transporter gene expression, we administered blocking antibodies against IL-6, TNF-α, or IFN-γ every second day. None of these treatments prevented the LCMV-induced downregulation of most BA transporters, with the exception of *Abcb11* (encoding BSEP), whose suppression was partially attenuated upon IL-6 and TNF-α blockade (Supplemental Figure 4, A–D). This is in line with previous reports indicating that TNF-α can regulate *Abcb11* expression in vitro (47), and suggests that IL-6 and TNF-α signaling modulates *Abcb11* expression during LCMV Cl13 infection.

To test whether classical cytotoxic function of CD8^+^ T cells may drive the downregulation of BA metabolism genes, we infected perforin-1–knockout mice (*Prf1^–/–^*) with LCMV Cl13. Loss of *Prf1* led to the expected decrease of hepatic injury as measured through ALT levels and also partly decreased other tissue damage markers including aspartate aminotransferase (AST) and lactate dehydrogenase (LDH) (Supplemental Figure 4E). Interestingly, *Prf1*-deficient mice only partially recovered the expression of BA metabolism genes (Supplemental Figure 4F), suggesting that additional factors may control their transcriptional regulation.

Taken together, our data demonstrate the role of adaptive immunity, particularly CD8^+^ T cells, in the transcriptional downregulation of hepatic BA transporters during LCMV Cl13 infection. Beyond cytokine expression and cytotoxicity of CD8^+^ T cells, additional CD8^+^-mediated mechanisms appear to modulate gene regulation of hepatic BA metabolism. These results further support our hypothesis that immune activation drives alterations in BA levels and BA transporter expression, highlighting the central role of CD8^+^ T cells in these changes.

### Pharmacological inhibition of NTCP increases hepatic damage without affecting CD8^+^ T cell responses.

LCMV Cl13 infection altered systemic BA metabolism and disrupted hepatic BA transporter expression, prompting us to assess whether the enterohepatic reuptake via NTCP and OATP1A/1B may affect CD8^+^ T cell responses. Upon infection, splenic CD8^+^ T cells upregulated the BA efflux transporter MDR1 (encoded by *Abcb1a* and *Abcb1b*), as well as *Slco3a1* (Supplemental Figure 5, A–C), which is consistent with findings from hepatocarcinoma models (48) and intestinal CD8^+^ T cells (49, 50) and suggests that CD8^+^ T cells sense the altered BA environment.

**Figure 4:**
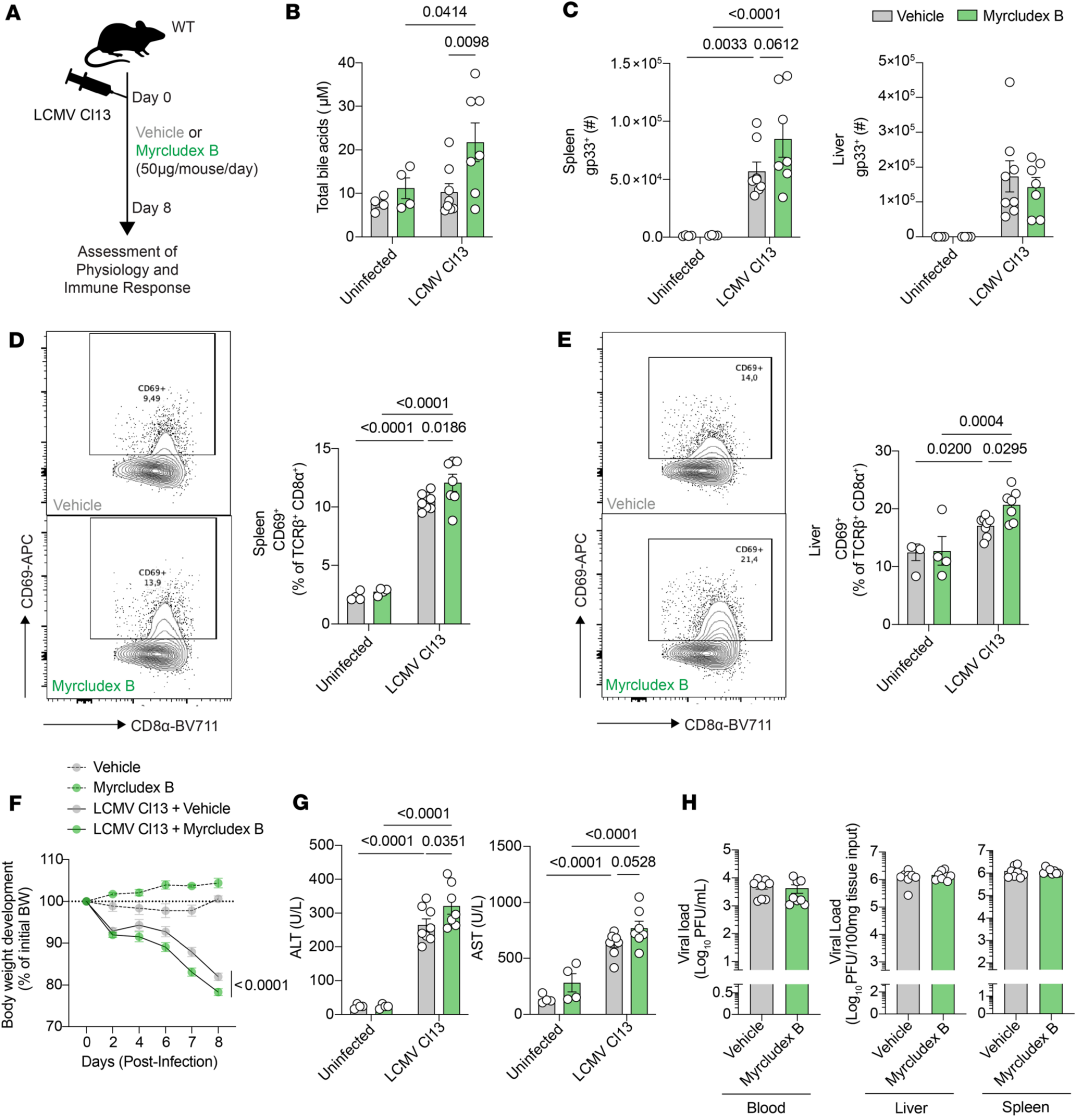
Inhibition of NTCP via systemic Myrcludex B administration increases hepatic damage, while having limited effects on the CD8^+^ T cell response. (**A**) Experimental setup. Mice were injected daily subcutaneously with 50 μg Myrcludex B over an 8-day time course. (**B**) Total BA levels in serum at day 8 after LCMV Cl13 infection. (**C**) Total CD8^+^ T cell numbers in liver and spleen at day 8 after LCMV Cl13 infection. (**D**) Frequency of CD69^+^ splenic CD8^+^ T cells at day 8 after infection. (**E**) Frequency of CD69^+^ CD8^+^ T cells in the liver at day 8 after infection. (**F**) Body weight development during LCMV Cl13 infection or in uninfected mice injected daily with either vehicle or Myrcludex B. (**G**) Blood levels of the hepatic damage markers ALT and AST at day 8 after LCMV Cl13 infection. (**H**) Viral loads in LCMV Cl13–infected mice upon vehicle or Myrcludex B treatment in blood, liver, and spleen at day 8 after infection. Data were pooled from 2 independent experiments (*n* = 4–8 mice per group) and analyzed using 2-way ANOVA with post hoc uncorrected Fisher’s LSD (**B**–**G**) or Mann-Whitney test (**H**).

Since LCMV infection shifted the BA pool toward conjugated host-derived species, we pharmacologically inhibited the conjugated BA transporter NTCP using Myrcludex B (Figure 4A) (7). Expectedly, NTCP inhibition boosted total serum BA levels upon infection with LCMV Cl13 (Figure 4B), potentially synergizing with the infection-induced downregulation of *Slco1a* and *Slco1b* transporters (51).

Next, we addressed the effects of these elevated BA levels on CD8^+^ T cell responses. Although total CD8^+^ T cell numbers in spleen and liver remained comparable upon LCMV Cl13 infection (Figure 4C), we found that CD8^+^ T cells in both organs had a modest but consistently higher expression of the activation and tissue residency marker CD69 (Figure 4, D and E). In contrast, NTCP inhibition showed no effect on CD8^+^ T cell proliferation or effector functions upon LCMV Cl13 infection (Supplemental Figure 6, A–C).

Given that LCMV Cl13 infection induces CD8^+^ T cell–dependent liver immunopathology, we next evaluated the pathophysiological effects of NTCP inhibition during LCMV Cl13 infection. Interestingly, NTCP inhibition led to a more severe body weight loss in mice infected with LCMV Cl13 (Figure 4F) and elevated levels of the hepatic tissue damage markers ALT and AST (Figure 4G). This indicated an aggravated hepatotoxicity, while viral titers remained comparable between the infected groups (Figure 4H). These findings suggest that NTCP inhibition exacerbates liver injury largely independently of the CD8^+^ T cell response. Collectively, our data indicate that functional loss of NTCP increases systemic BA levels and hepatic injury, while having limited impact on the underlying CD8^+^ T cell response.

### CD8^+^ T cell response is impaired in mice with genetic loss of Slco1a/1b BA transporters.

**Figure 5:**
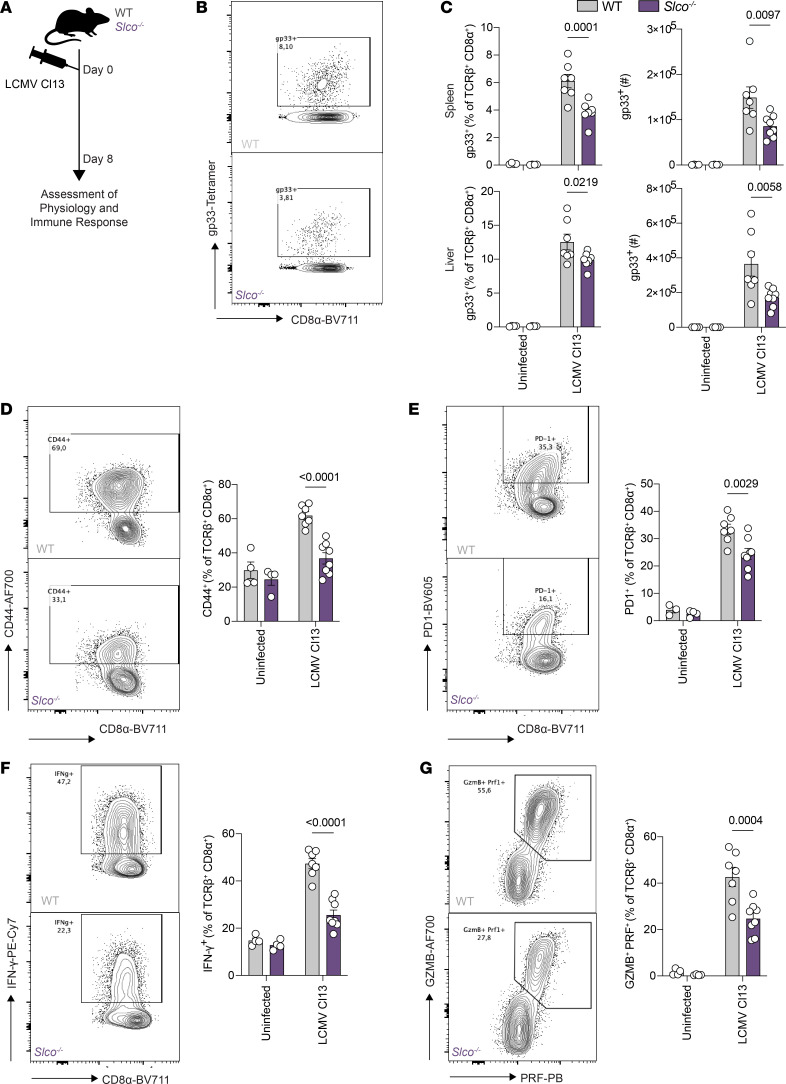
Impaired splenic CD8^+^ T cell response to LCMV Cl13 infection in mice with systemically elevated BAs. (**A**) Schematic of experimental design of LCMV infection in *Slco^–/–^* mice. (**B**) Representative flow cytometry plot of LCMV GP33–specific CD8^+^ T cells in the spleen 8 days after LCMV Cl13 infection. (**C**) Frequency and total number of LCMV GP33–specific CD8^+^ T cells in spleen and liver at day 8 after infection. (**D** and **E**) Frequency of CD44-expressing (**D**) and PD-1–expressing (**E**) CD8^+^ T cells in the spleen at day 8 after LCMV Cl13 infection. (**F**) Frequency of IFN-γ–producing splenic CD8^+^ T cells after 4 hours of restimulation with PMA and ionomycin at 8 days after infection. (**G**) Frequency of GZMB^+^PRF^+^ splenic CD8^+^ T cells at day 8 after LCMV Cl13 infection. Data were pooled from 2 independent experiments (*n* = 4–8 mice per group) and analyzed using 2-way ANOVA with post hoc Šidák’s multiple-comparison test (**C**–**G**).

Next we sought to test the consequences of loss of unconjugated BA transporter family *Slco1a/1b* (hereafter referred to as *Slco^–/–^*), which results in systemically high levels of unconjugated BAs (10). First, we confirmed the deletion of genes involved in the cassette, including *Slco1a1* in the liver (Supplemental Figure 7A). As previously described (10), this deletion led to elevated levels of total serum BAs (Supplemental Figure 7B) and bilirubin (Supplemental Figure 7C) in uninfected mice. Following LCMV Cl13 infection (Figure 5A), *Slco^–/–^* mice showed a decrease in virus-specific CD8^+^ T cells in the spleen compared with littermate controls (Figure 5, B and C), despite having comparable total CD8^+^ T cell numbers in the spleen (Supplemental Figure 7D). Interestingly, upon infection, splenic CD8^+^ T cells in *Slco^–/–^* mice displayed reduced expression of the activation markers CD44 and PD-1 (Figure 5, D and E) and expression of the cellular proliferation marker Ki-67^+^ (Supplemental Figure 7E), indicating an impaired activation and proliferation of splenic CD8^+^ T cells in *Slco^–/–^* mice.

Next, we tested whether this resulted in reduced functionality. Upon PMA/ionomycin restimulation, total CD8^+^ T cells from *Slco^–/–^* mice produced less IFN-γ (Figure 5F), and fewer cells expressed the cytolytic proteins granzyme B (GZMB) and perforin (PRF) (Figure 5G). These findings align with the reduced activation and presence of virus-specific CD8^+^ T cells in the spleen of *Slco^–/–^* mice. However, antigen-specific restimulation with immunodominant viral epitope GP_33–41_ showed no differences in the production of effector molecules among GP33-specific CD8^+^ T cells (Supplemental Figure 7F), suggesting that virus-specific CD8^+^ T cells in *Slco^–/–^* mice are functional upon encountering their cognate antigen, but suffer from impaired activation and expansion.

Consistent with our results in the spleen, we found a reduction in LCMV-specific CD8^+^ T cells in the liver (Figure 5C), which was also accompanied by a reduction in total CD8^+^ T cells (Supplemental Figure 8A). This led to a reduction in total numbers of activated CD8^+^ T cells (Supplemental Figure 8, B and C). Interestingly, CD8^+^ T cells of *Slco^–/–^* mice produced cytokines and cytolytic proteins similarly to CD8^+^ T cells of their littermate controls upon restimulation (Supplemental Figure 8D), indicating that liver-infiltrating CD8^+^ T cells are reduced in number, but functional.

To address whether the impaired CD8^+^ T cell response was lasting, we quantified CD8^+^ T cells in LCMV Cl13–infected *Slco^–/–^* mice and littermate controls at day 12 after infection. At this time point, we found that the CD8^+^ T cell response in *Slco^–/–^* mice had largely caught up to levels similar to those in littermate controls in both spleen and liver (Supplemental Figure 9, A and B). Yet, splenic virus-specific CD8^+^ T cells as well as the frequency of CD8^+^ T cells expressing CD44^+^ and effector molecules (IFN-γ^+^ and GZMB^+^PRF^+^) still tended to be reduced in *Slco^–/–^* mice (Supplemental Figure 9A).

Taken together, these results indicate that *Slco1a/1b* ablation impairs CD8^+^ T cell activation and expansion in the spleen and delays virus-specific T cell accumulation, leading to overall reduced effector response.

### Loss of BA transporter family Slco1a/1b attenuates liver damage.

**Figure 6:**
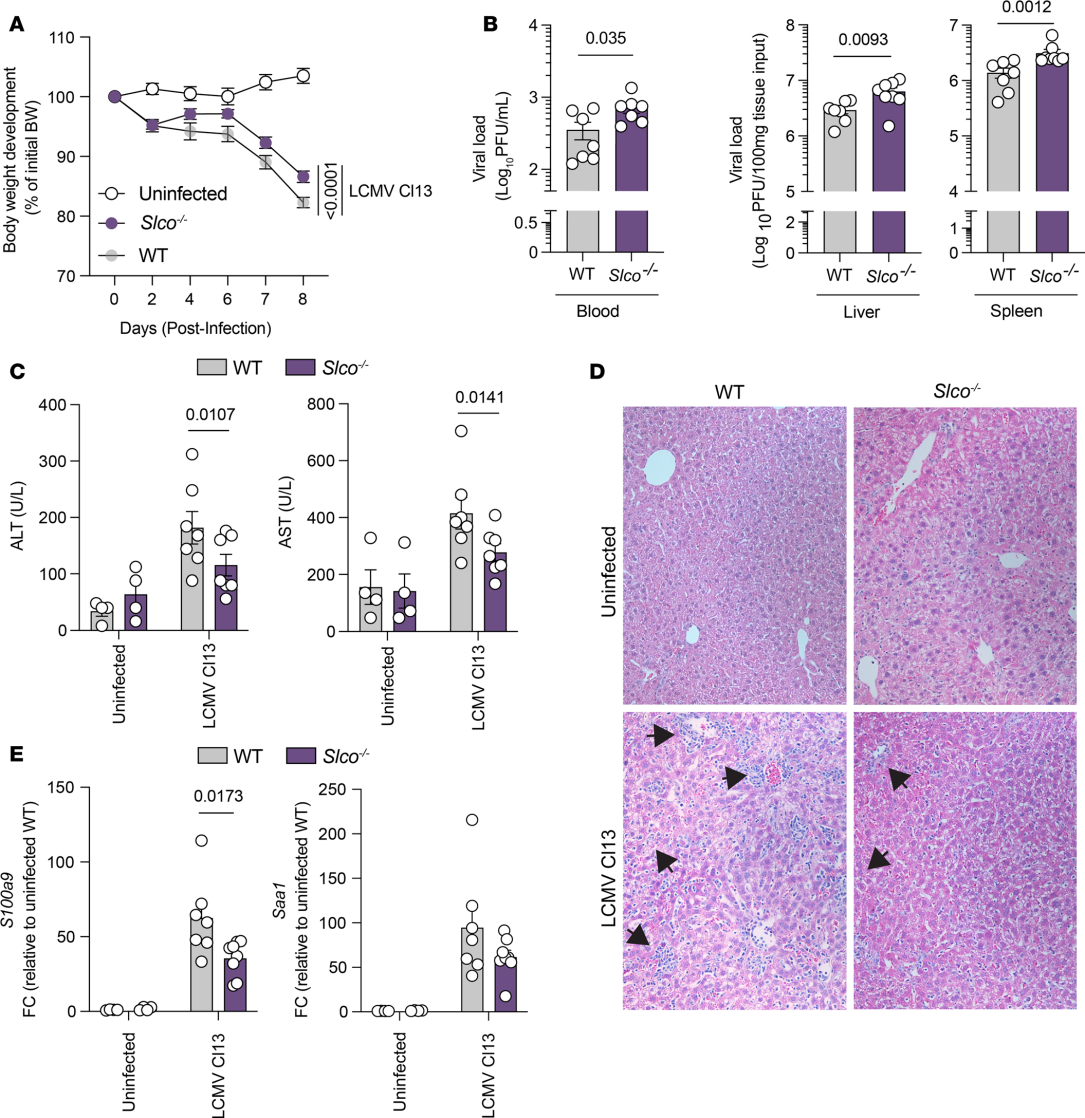
Loss of the BA transporters *Slco1a* and *Slco1b* reduces T cell–mediated liver damage during LCMV Cl13 infection. (**A**) Body weight development of *Slco^–/–^* mice or littermates infected with LCMV Cl13. (**B**) LCMV Cl13 titers in blood, liver, and spleen at 8 days after infection in *Slco^–/–^* mice or littermate controls. (**C**) Serum ALT and AST levels in *Slco^–/–^* mice or littermate controls at 8 days after infection. (**D**) Representative H&E staining of liver sections from WT or *Slco^–/–^* mice at day 8 after infection or left uninfected. Arrows indicate cellular infiltrates. Original magnification: ×20. (**E**) Hepatic gene expression of acute-phase proteins in *Slco^–/–^* mice or littermate controls at 8 days after infection. Data were pooled from 2 independent experiments (*n* = 4–8 mice per group) and analyzed using 2-way ANOVA (**A**), using 2-way ANOVA blocked for the experiment (**C** and **E**), or using 2-tailed Mann-Whitney *t* test (**B**).

Based on the observed impairment in CD8^+^ T cell activation in the *Slco^–/–^* mouse model, we wanted to assess its impact on pathophysiology and pathogen load upon viral infection. *Slco^–/–^* mice infected with LCMV exhibited a delayed onset of body weight loss compared with littermate controls (Figure 6A). Based on the reduced virus-specific T cell response, we hypothesized that viral clearance may be affected at day 8 after infection. Indeed, *Slco^–/–^* mice displayed increased viremia, and higher viral loads in the liver and spleen (Figure 6B). Consistent with the reduced T cell response and increased viremia, we found that genetic ablation of *Slco1a/1b* transporters led to reduced levels of the liver damage markers ALT and AST (Figure 6C). Histological assessment of liver pathology showed a decreased presence of cellular infiltrates (Figure 6D), which is in line with a decreased presence of virus-specific CD8^+^ T cells in the liver of *Slco^–/–^* mice infected with LCMV Cl13 (Figure 5C). To confirm the altered inflammatory state of the liver, we assessed the expression of acute-phase proteins and found that these were slightly reduced in *Slco^–/–^* mice (Figure 6E). At a later time point, *Slco^–/–^* mice caught up to comparable levels of viremia and hepatic damage compared with littermate controls (Supplemental Figure 9, C–G). This is in line with the expansion of their CD8^+^ T cell compartment to levels similar to those in littermate controls (Supplemental Figure 9, A and B). Together, our data suggest that loss of *Slco1a/1b* substantially delays CD8^+^ T cell responses and thereby prevents initial CD8^+^ T cell–driven immunopathology, possibly as a result of the high unconjugated BA levels.

## Discussion

In this study, we investigated the interplay between BA metabolism and adaptive immunity, demonstrating that CD8^+^ T cell–mediated hepatitis leads to a downregulation of BA metabolism genes, while also increasing systemic BA levels and shifting BA composition. Pharmacological inhibition of the conjugated BA transporter NTCP elevated systemic BA levels and exacerbated liver injury while CD8^+^ T cell responses remained largely unaffected. In contrast, mice with genetic ablation of *Slco1a/1b* transporters as a model of sustained systemic elevation of unconjugated BAs exhibited impaired CD8^+^ T cell responses and a concomitant decrease in CD8^+^ T cell–mediated immunopathology and viral clearance. These findings highlight a crosstalk between CD8^+^ T cells and systemic BA metabolism that alters antiviral immunity and/or liver pathology.

Liver diseases substantially affect systemic BA levels (52–57). Consistent with findings in LCMV WE, LCMV Cl13 infection increased levels of BAs in the circulation (37). An in-depth BA analysis further highlighted a shift toward host-derived and conjugated BAs, akin to patients with hepatitis B (21, 54). Comparable accumulation of conjugated BAs, including T-CA, T-βMCA, and T-CDCA, has also been reported in hepatocarcinoma patients and mouse models (48). The compositional shift in BA species during LCMV Cl13 infection may be explained by the downregulation of hepatic BA reuptake transporters, such as *Slc10a1*, which may increase systemic BA levels of host-derived, conjugated BAs. The undisrupted expression of *Baat*, the sole enzyme responsible for conjugating BAs (4), further supports this compositional shift. Similar patterns of reduced BA transporter expression with preserved *BAAT* levels have been described in chronic hepatitis B (21), while hepatocarcinoma is associated with *BAAT* overexpression and enhanced conjugated BA accumulation (48). Additionally, expression of *Cyp2a12* remained unchanged during LCMV Cl13 challenge, supporting the conversion of deoxycholic acid (DCA) into cholic acid (CA), thereby enriching for host-derived BA species (58). Lastly, leakage of host-derived conjugated BAs from damaged hepatocytes may also contribute to this compositional shift. Together, these parallels support LCMV infection as a pathophysiologically relevant model to study BA metabolism in liver disease.

Additionally, liver diseases, such as cholestatic diseases and viral hepatitis, show sustained repression of genes involved in BA metabolism (59, 60). While the FGF15/FXR/SHP axis is the main driver of this downregulation in cholestatic diseases through the physiological feedback inhibition by BAs in the gut (59), our data indicate that this pathway is unlikely to be involved in the downregulation of BA transport and synthesis genes in the context of LCMV-induced hepatitis. In addition, increased BA levels may activate additional pathways such as FGFR4–MAPK/ERK signaling, which in turn controls BA metabolism gene expression. While this cannot be fully excluded, our data highlight that the adaptive immune response, specifically CD8^+^ T cells, appears to mediate transcriptional downregulation directly.

Inflammatory cytokines such as IL-6, IL-1β, and TNF-α have been reported to control BA transporter expression (13, 61, 62); however, in our model, IL-6, TNF-α, and IFN-γ were largely dispensable for the LCMV-induced downregulation of BA transporters, with the exception of *Abcb11*/BSEP. In contrast, CD8^+^ T cell–mediated cytotoxicity partially restored several BA genes, suggesting that hepatocyte killing may directly or indirectly drive the liver-specific suppression of BA metabolism genes during LCMV Cl13 infection. It remains clear, however, that CD8^+^ T cells are critical for suppression of BA metabolism genes through cytotoxic and yet unknown effects.

Recently, it has become clear that CD8^+^ T cells are highly sensitive to environmental BA levels (48–50). In LCMV Cl13–infected wild-type mice, splenic CD8^+^ T cells upregulate various BA efflux transporters, including *Abcb1a/1b* and *Slco3a1*, suggesting that they respond to the altered BA environment. Although disruption of the enterohepatic cycle commonly elevates systemic BAs, its impact on CD8^+^ T cells has remained unclear. We observed distinct outcomes following pharmacological inhibition of NTCP versus genetic deletion of *Slco1a/1b*, which mediate hepatic uptake of conjugated and unconjugated BAs, respectively. NTCP inhibition resulted in increased hepatotoxicity, consistent with reports that Myrcludex B treatment can aggravate body weight loss and liver injury in certain cholestatic models (63), but had limited effects on the CD8^+^ T cell response — possibly owing to compensatory functions of *Slco1a/1b* (7). In contrast, *Slco1a/1b* deficiency severely impaired CD8^+^ T cell activation, reducing viral clearance and immunopathology. Potential mechanisms underlying the impaired CD8^+^ T cell response in *Slco^–/–^* mice include direct effects of BA species on Ca^2+^ (31, 64) or mTOR signaling (33), indirect modulation of dendritic cell cross-presentation (29), and impaired T cell homing to the liver. However, the reduction in virus-specific T cell numbers and activation in the spleen argues against defective liver homing as a primary cause.

It cannot be ruled out that specific BA species or their combination are responsible for the immunomodulatory effect seen in *Slco^–/–^* mice. In addition to host-derived BAs, microbiota-derived secondary BAs such as lithocholic acid (LCA) and its derivatives (e.g., 3-oxo-LCA and 3-isoallo-LCA) have been reported to modulate adaptive immune responses (27–29). During LCMV Cl13 infection, only marginal changes in serum LCA levels were observed. It remains unclear which exact BA may be causing the suppressive effects on the CD8^+^ T cell response, which could include more localized effects of microbiota-derived BA species.

Using *Slco^–/–^* mice as a model of systemically high BA levels comes with additional effects, including elevated systemic bilirubin and altered energy metabolism (10). In addition, some of the genes ablated in *Slco^–/–^* mice have roles in other organs, such as the brain and kidney (9), complicating the establishment of clear causal relationships in the *Slco^–/–^* model.

In conclusion, LCMV-induced hepatitis perturbs BA metabolism in a manner resembling human liver diseases (4). The resulting alterations may have immunomodulatory potential to modulate CD8^+^ T cell–mediated immunopathology. These findings underscore the role of systemic BA metabolism in shaping immunity during liver diseases and support further investigations of the crosstalk between BA metabolism and the immune system as a potential therapeutic avenue.

## Methods

### Sex as a biological variable.

Both male and female mice were used for this study. Sex-specific stratifications were not performed.

### Mice.

Unless otherwise stated, mice were 8–15 weeks old at the start of experiments, and all mice were age- and sex-matched within experiments.

Animals were kept and bred at the Core Facility Laboratory Animal Breeding and Husbandry of the Medical University of Vienna, under specific pathogen–free conditions. Mouse experiments were conducted in individual ventilated cages in compliance with the animal experiment licenses BMWFW-66.009/0361-WF/V/3b/2017, 2020-0.406.011, and 2024-0.363.541, approved by the institutional ethical committee of the Department for Biomedical Research of the Medical University of Vienna.

Wild-type mice on C57BL/6J background were bred in-house or purchased via Janvier Labs (catalog 0006). *Slco1a1b^–/–^* mice were obtained from Alfred Schinkel (Netherlands Cancer Institute, Amsterdam, Netherlands) on an FVB background. These mice were backcrossed onto C57BL/6J for 6 or more generations. For backcrossed mice, we used heterozygous or wild-type littermates as controls.

Other genetically modified animals were on C57BL/6 background and purchased via The Jackson Laboratory: *Ifnar1^–/–^* (65) (032045-JAX), *Rag2^–/–^* (46) (008449-JAX), *B2m^–/–^* (66) (002087-JAX), and *Prf1^–/–^* (67) (002407-JAX). Pair-feeding experiments were performed on individually housed mice to ensure precise measurement of food intake. They adapted to solitary housing for 3 days before infection. Infected mice then received food ad libitum, while pair-fed counterparts were given the same exact amount of food consumed by infected mice.

### Infections.

Mice were infected intravenously with 2 × 10^6^ focus-forming units of lymphocytic choriomeningitis virus clone 13 strain (LCMV Cl13). LCMV Cl13 was grown in BHK-21 cells, and viral titers were determined with focus-forming assay (68). Mice were sacrificed at the indicated time points, and tissues were snap-frozen in liquid nitrogen to be stored at –80°C until further analysis.

### Treatment with blocking antibodies and NTCP inhibitor.

For the blocking of cytokines, animals received 0.5 mg/mouse of the following antibodies: anti–IL-6 (MP5-20F3, rat IgG1, Bio X Cell, BE0046), anti–IFN-γ (XMG1, rat IgG1, Bio X Cell, BE0055), anti–TNF-α (XT3.110, rat IgG1, Bio X Cell, BE0058), or a rat IgG1 isotype (MOPC-21, Bio X Cell, BE0083). The injections were given intraperitoneally every other day for 7 days, with the first given 1 day before the infection with LCMV Cl13.

For CD8^+^ T cell depletion, animals received 0.2 mg/mouse of anti-CD8 (YTS169.4, rat IgG2b, Bio X Cell, BE0117) or a rat IgG2b isotype (LTF-2, Bio X Cell, BE0090). The injections were given intraperitoneally 2 days and 1 day before infection with LCMV Cl13.

The NTCP inhibitor Myrcludex B (Sigma-Aldrich, SML3515-5MG) was prepared in a stock concentration of 500 μg/mL in the vehicle solution (25 mM sodium carbonate, 50 mg/mL d-mannitol, pH ~8.8), as previously described (69). Treated mice received daily subcutaneous injections of 100 μL Myrcludex B (50 μg/mouse/day), while control mice received injections of vehicle solution daily. Treatment was started on the day of LCMV Cl13 infection and done daily until day 8 after infection.

### Blood chemistry.

For serum analysis, blood samples were centrifuged at 10,000*g* for 5 minutes at 4°C, after which serum was stored in a new tube at –80°C until further analysis. On the day of the analysis, serum samples were diluted 1:8 in PBS. Alanine aminotransferase (ALT), aspartate aminotransferase (AST), albumin, bilirubin, and cholesterol were measured using a Cobas C311 analyzer (Roche). Total BAs in the serum were determined by an enzymatic assay kit (Cell Biolabs, STA-631; or DiaSys, 1 2238), according to the manufacturer’s instructions.

### RNA isolation and real-time PCR.

The TissueLyser II (Qiagen) was used to homogenize the tissues, and total RNA extraction was performed using Qiazol Lysis Reagent (Qiagen, 79306) following the manufacturer’s instructions. cDNA was synthesized using the RevertAid First Strand cDNA Synthesis Kit (Thermo Fisher Scientific, K1622). Real-time PCR was performed using a TaqMan Fast Universal PCR Master Mix (Thermo Fisher Scientific, 4352042), and TaqMan Gene Expression Assays (Thermo Fisher Scientific) against the following mouse gene products: Slco1a1 (Mm00649796_m1), Slco1b2 (Mm00451510_m1), Abcb11 (Mm00445168_m1), Slc10a1 (Mm00441421_m1), Nr1h4 (Mm00436425_m1), Cyp7a1 (Mm00484150_m1), Cyp2c70 (Mm00521058_m1), Abcb1a (Mm00440761_m1), Abcb1b (Mm00440736_m1), Nr0b2 (Mm00442278_m1), Saa1 (Mm00656927_g1), S100a9 (Mm00656925_m1), Fgf15 (Mm00433278_m1), and Slc10a2 (Mm00488258_m1). Ef1a was also measured by TaqMan chemistry using the primers 5′-GCAAAAACGACCCACCAATG-3′ and 5′-GGCCTTGGTTCAGGATA-3′ and probe 5′-[6FAM]CACCTGAGCAGTGAAGCCAG[TAM]-3′.

### Isolation of intrahepatic lymphocytes.

Intrahepatic lymphocytes were assessed as previously described (70). In brief, livers were perfused via the portal hepatic vein using cold PBS. The gallbladder was removed, and the livers were collected in cold PBS. Tissue was mechanically disrupted using a 70 μm cell strainer (Sarstedt) to obtain a single-cell suspension. Cells were pelleted at 400*g* for 5 minutes at 4°C. Cells were then resuspended in a 42% Percoll solution (Cytiva, 17-0891-02) and centrifuged for 20 minutes, 800*g*, at room temperature without brake. Red blood cells were lysed by resuspension of the pellet in 1 mL of RBC Lysis Buffer (Invitrogen, 00-4333-57) for 3 minutes at room temperature. Cells were pelleted at 400*g* for 5 minutes at 4°C. Finally, cells were resuspended, and flow cytometry staining was performed.

### Flow cytometry.

Spleens were isolated and collected in cold PBS. Single cells were isolated by mechanical disruption of spleens through a 70 μm cell strainer (Sarstedt). Cells were spun down at 400*g* for 5 minutes at 4°C. Pellets were resuspended in 1 mL of 1× RBC Lysis Buffer (Invitrogen, 00-4333-57) for 5 minutes at room temperature. Cells were washed in PBS and spun down again at 400*g* for 5 minutes at 4°C. Cells were resuspended in cold PBS and used for flow cytometry staining. For PMA/ionomycin restimulation, cells were plated in 96-well plates and then restimulated with Cell Activation Cocktail with Brefeldin A (BioLegend, 423303) for 4 hours at 37°C. For viral peptide restimulation, cells were initially incubated with the peptide alone for 30 minutes at a concentration of 1 μg/mL at 37°C. Subsequently, at the same peptide concentration, Protein Transport Inhibitor Cocktail (eBioscience, 00-4980-03) was added, and cells were incubated at 37°C for 4 hours. After incubation, cells were washed and stained for surface markers and intracellular proteins.

For surface staining, cells were incubated with fluorophore-labeled antibodies, Fixable Viability Dye eFluor 780 (eBioscience, 65-0865-14), and CD16/32 FcR-Block (BioLegend, 101302). In the case of virus-specific tetramer staining (i.e., PE-labeled GP33-specific tetramers acquired from the US NIH Tetramer Core Facility), cells were incubated for staining at room temperature for 20 minutes. After being washed twice, cells were fixed in 4% paraformaldehyde for 10 minutes at room temperature. In the case of intracellular staining, cells were fixed/permeabilized using the eBioscience Foxp3/Transcription Factor Staining Set (00-5523-00, Invitrogen). Cells were incubated with intracellular staining overnight at 4°C. The following antibodies were used for the staining: TCRb-PerCP-Cy5.5 (BioLegend, 109227), CD8a-BV711 (BioLegend, 100747), CD44-AF700 (BioLegend, 103025), granzyme B–AF700 (BioLegend, 372221), perforin-PB (BioLegend, 154311), TNF-α–APC (BioLegend, 506307), IFN-γ–PE–Cy7 (BioLegend, 505826), PD-1–BV605 (BioLegend, 135219), Ki-67-AF488 (BioLegend, 151204), and CD69-APC (BioLegend, 104513). An exemplified gating strategy is provided (Supplemental Figure 10).

### Targeted LC-MS–based metabolite measurements.

Profiles of murine primary and secondary unconjugated and conjugated C24-BAs in serum (30 μL) were analyzed as published previously (71).

### DNA extraction, library preparation, and 16S rRNA sequencing.

Four-week-old female C57BL/6J mice were used and were cohoused in our mouse facility. The mice were randomly mixed together, and bedding was exchanged between cages twice a week for a period of 4 weeks to maximize normalization of microbiome across animals. Mice were infected at 8 weeks old and harvested at the indicated days after infection. Instruments were cleaned and disinfected between mice. Tissues and tissue contents were snap-frozen in liquid nitrogen immediately after collection and stored at –80°C before being handed over to the joint microbiome facility. DNA was extracted using the QIAamp Fast DNA Stool Mini Kit following the manufacturer’s protocol, automated on a QIACube Connect Instrument (Qiagen). Sample lysis in lysis buffer was performed by bead beating, twice for 60 seconds at 14,000 rpm with a FastPrep-96 instrument (MP Biomedicals). The V4 hypervariable region of the bacterial and archaeal 16S rRNA gene was amplified using primers 515F and 806R (72, 73), barcoded following the protocol described by Pjevac et al. (74), and sequenced on the Illumina Miseq Platform (V3 chemistry, 600 cycles) at the Joint Microbiome Facility of the Medical University of Vienna and the University of Vienna, under project ID JMF-2202-03. Data are available under NCBI BioProject ID PRJNA1425465.

### Ex vivo BSH activity measurement.

For the analysis of BSH activity from cecal-content protein extracts, we used a published protocol (75). Briefly, harvested cecal content was mixed with protease inhibitor (cOmplete, Sigma-Aldrich) and 1 mM DTT at 100 mg/ mL per sample. Samples were lysed in Lysing Matrix E tubes (MP Biomedicals) and homogenized in a Precellys homogenizer (3 × 30 seconds, 6,000 rpm, with 2-minute pause on ice), followed by centrifugation (20,000*g*, 30 minutes at 4°C). Supernatant was filtered with a 0.25 filter and concentrated using Amicon 10 kDa cartridges (Millipore). Protein concentration was measured using bicinchoninic acid, and 100 μg of protein was combined with 5 mM TDCA and 5 mM DTT in PBS (pH 5.6). BSH activity was assessed via OD_600_ of precipitated DCA, against a DCA standard row.

### Bioinformatics analysis.

For the analysis of bile acid LC-MS measurements, the measured BA concentrations (nmol/g) were normalized by 100 × *c*/sum(*c*), *c* being the measured concentration. The relative BA abundances obtained in this way were averaged across the replicates. BAs that were not in the top 9 BAs with respect to their peak relative abundance were aggregated into the “other” category. The relative abundances across time were visualized using a bump plot, depicting the relative share of each BA and its rank across time.

Heatmaps were made with the ComplexHeatmap package (76), and KEGG pathway enrichments were performed using the clusterProfiler package (77) in R version 4.2.2. For the analysis of the 16S rRNA dataset, Amplicon pools were extracted from the raw sequencing data using the FASTQ workflow in BaseSpace (Illumina) with default parameters. Raw data processing was performed as described previously (74). Demultiplexing was performed with the Python package demultiplex (Laros JFJ, github.com/jfjlaros/demultiplex) allowing 1 mismatch for barcodes and 2 mismatches for linkers and primers. Amplicon sequence variants (ASVs) were inferred using the DADA2 R package v1.24 (78) applying the recommended workflow (79). FASTQ reads 1 and 2 were trimmed at 220 nt and 150 nt, respectively, with allowed expected errors of 2. Relative abundance of bacterial bile salt hydrolase genes (EC 3.5.1.24) in feces and cecal content of uninfected or LCMV Cl13–infected mice 8 days after infection was predicted with PICRUSt2 (80) implemented in QIIME2 (81).

### Statistics.

The results are presented in the format of mean ± SEM and were subjected to statistical analysis as specified in the figure legends, using GraphPad Prism. Relevant *P* values are presented. A *P* value of less than 0.05 was considered significant. Unless otherwise specified in the figure legend, data show representative results from at least 2 independent experiments. For pooled data, owing to the smaller individual experimental group size, statistical analysis may be blocked for the experiment.

### Study approval.

Mouse experiments were conducted in individual ventilated cages in compliance with the animal experiment licenses BMWFW-66.009/0361-WF/V/3b/2017, 2020-0.406.011, and 2024-0.363.541, approved by the institutional ethical committee of the Department for Biomedical Research of the Medical University of Vienna.

### Data availability.

The metabolomics dataset used to support our findings in Figure 1B is available as Supplemental Table 1. A datasheet with the relevant expression values for Figure 2B is provided in Supplemental Figure 2. The 16S rRNA sequencing dataset is available under NCBI BioProject ID PRJNA1425465. A Supporting Data Values file is available in accordance with *JCI Insight* policy.

## Author contributions

FCR and ZK performed conceptualization, formal analysis, investigation, and visualization, developed methodology, wrote the original draft of the manuscript, and reviewed and edited the manuscript. CV performed investigation and formal analysis and reviewed and edited the manuscript. MB performed investigation, developed methodology, and reviewed and edited the manuscript. HGC, MS, LH, and HB performed investigation and reviewed and edited the manuscript. AH, A Burrett, and AS performed investigation. LE performed formal analysis. JXEW performed investigation. LAH performed investigation and reviewed and edited the manuscript. OP performed formal analysis. FA performed formal analysis and reviewed and edited the manuscript. JWG performed formal analysis. CDF performed investigation and reviewed and edited the manuscript. HS and HUM performed investigation and conceptualization. TR performed conceptualization and reviewed and edited the manuscript. KSL performed conceptualization. CC and MT performed conceptualization and reviewed and edited the manuscript. A Bergthaler performed supervision, project management, and conceptualization and reviewed and edited the manuscript.

## Conflict of interest

The authors have declared that no conflict of interest exists.

## Funding support

- European Union’s Horizon 2020 research and innovation program under European Research Council grant agreement 677006 (CMIL; to A Bergthaler).
- Marie Skłodowska-Curie Actions (MSCA) Innovative Training Network H2020-MSCA-ITN-2019 grant agreement 813343 (INITIATE; to A Bergthaler) and grant agreement 101028971 (Ammoniavir; to HGC).
- Austrian Academy of Sciences under the Disruptive Innovation program grant agreement DI-2023-093 (Cachexia-DeRBy; to FCR) and DOC fellowships (to HB and JWG).
- Austrian Science Fund (FWF) (Cluster of Excellence “Microbiomes Drive Planetary Health” [10.55776/COE7]) grants P35806 and P36646 (to A Bergthaler).

## Supplementary Material

Supplemental data

Supplemental table 1

Supplemental table 2

Supporting data values

## Version 1 06/22/2026

Electronic publication

## Version 2 06/24/2026

graphical abstract added
