# Supplementary material for: Crosstalk between CD8+ T cells and systemic bile acid metabolism shapes antiviral immunity and immunopathology
Source: JCI Insight. 2026 Jun 22;11(12):e189882. doi: 10.1172/jci.insight.189882 (PMC13313544; doi:10.1172/jci.insight.189882)
Supplement: Supplemental data [file jciinsight-11-189882-s336.pdf]

Supplementary figures

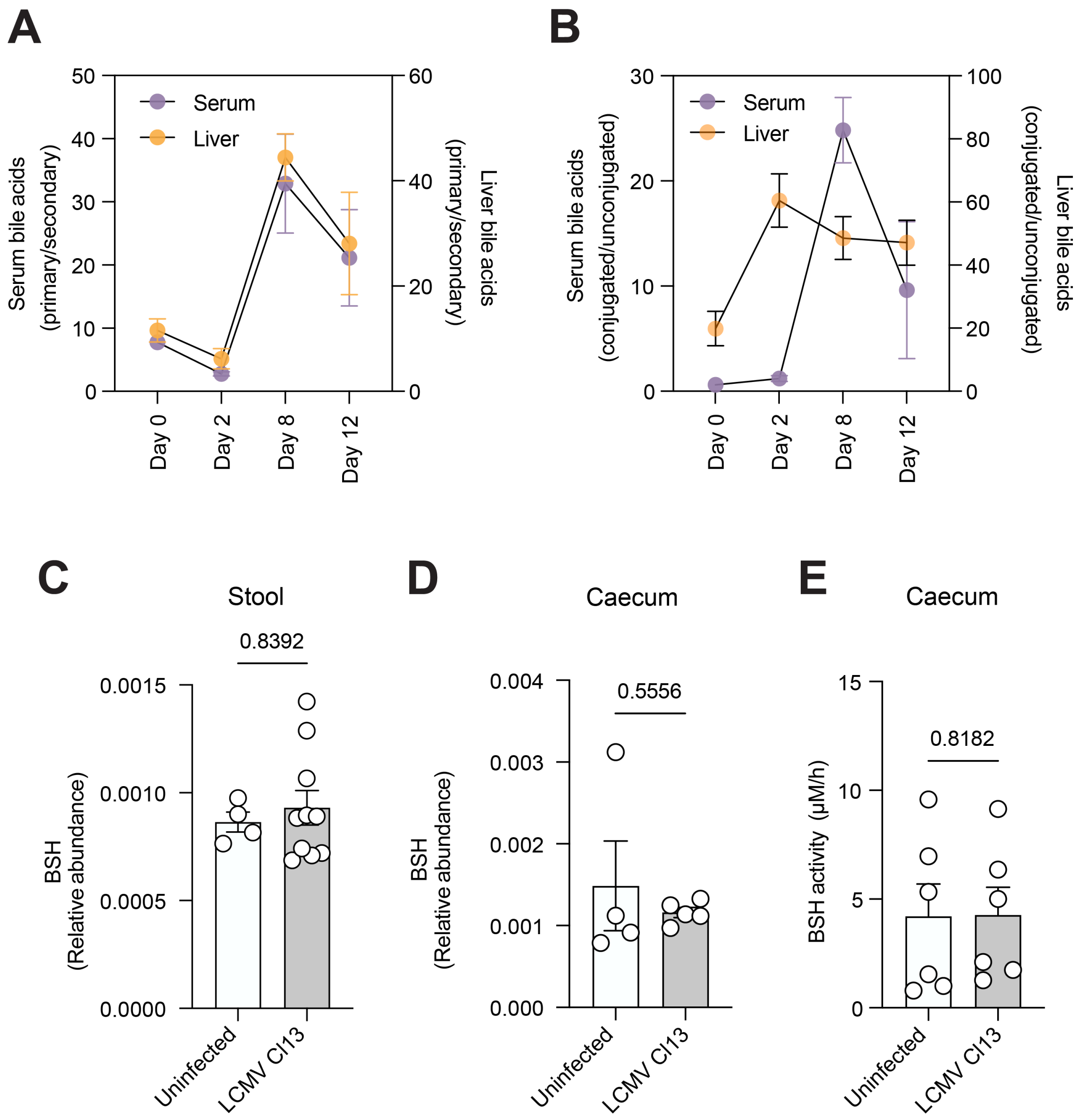

Supplementary Figure 1: Chronic LCMV infection alters BA composition, presumably independent of BSH activity. (A) Ratio of host-to-microbe-derived BA species in serum and liver over the course of LCMV infection. Data representative from two independent experiments (n = 3-4 mice/group). (B) Ratio of conjugated-to-unconjugated BA species in serum and liver over the course of LCMV infection. Data representative from two independent experiments (n = 3-4 mice/group). (C) Relative abundance of BSH in 16S sequencing dataset of stool samples from mice upon LCMV Cl13 infection collected at day 8 post-infection. Data

pooled from one independent experiments (n = 4-10 mice/group) and analyzed using Mann-Whitney test. (D) Relative BSH abundance in caecum at day 8 post-LCMV infection. Data pooled from one independent experiments (n=4-5 mice/group) and analyzed using Mann-Whitney test. (E) BSH activity measurement in caecum measured at day 8 post-infection. Data pooled from two independent experiments (n=6 mice/group) and analyzed using Mann-Whitney test.

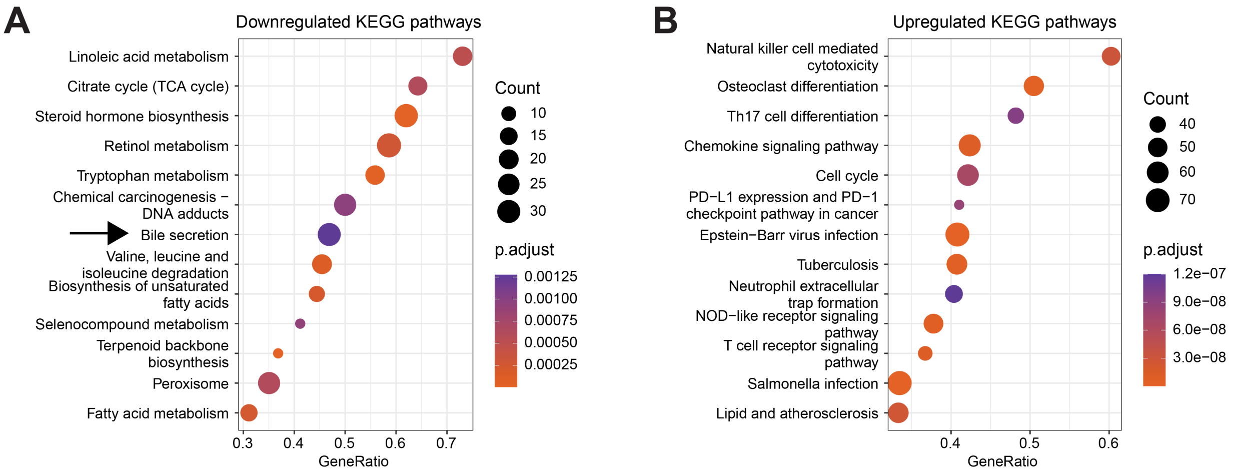

Supplementary Figure 2: Pathway enrichment analysis of livers in response to LCMV Cl13 infection. (A,B) Gene set enrichment analysis of hepatic gene expression dataset (42) at day 8 post-LCMV Cl13 infection for downregulated (A) and upregulated (B) KEGG pathways.

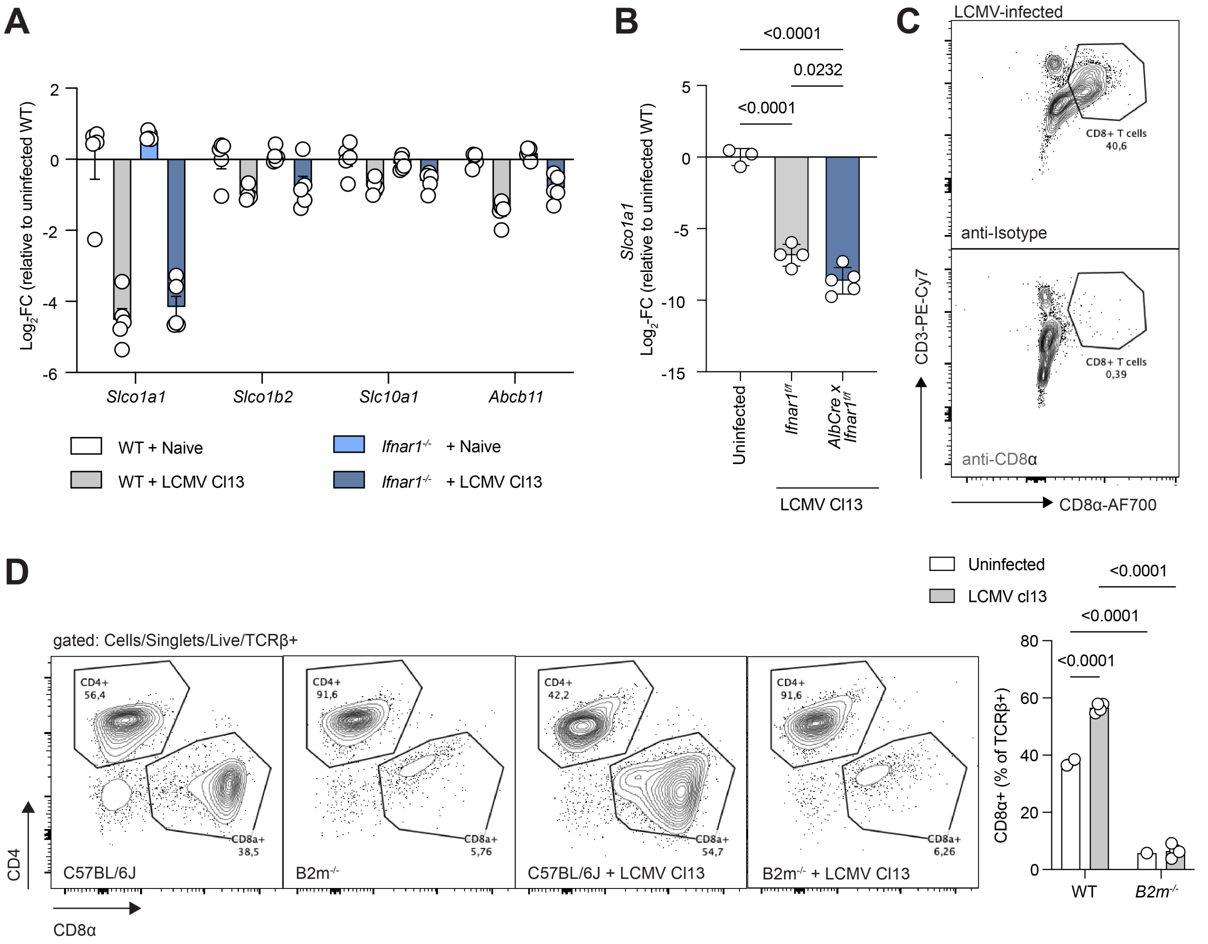

Supplementary Figure 3: Downregulation of bile acid transporters in the liver is independent of type I signaling and dependent on CD8$^+$ T cells. (A) Expression of BA transporters in *Ifnar*-deficient mice at 8 days post LCMV Cl13 infection. Data representative for two independent experiments (n = 5 mice/group) and analyzed using multiple unpaired t-test. (B) *Slco1a1* expression in liver-specific *Ifnar*-deficient mice at 8 days post LCMV Cl13 infection. Data representative for two independent experiments (n = 3-5 mice/group) and analyzed using One-Way ANOVA with post-hoc Tukey's multiple comparison. (C) Representative FACS plots of CD8$^+$ T cell depletion upon anti-CD8a treatment at day 8 post-LCMV Cl13 infection in the spleen. (D) Representative FACS plots and quantification of CD8$^+$ T cell depletion in *B2m*-deficient mice (*B2m*$^{-/-}$) at day 8 post-LCMV Cl13 infection in the spleen. Data representative for three independent experiments (n = 2-4 mice/group) and analyzed using Two-Way ANOVA with post-hoc uncorrected Fisher's LSD.

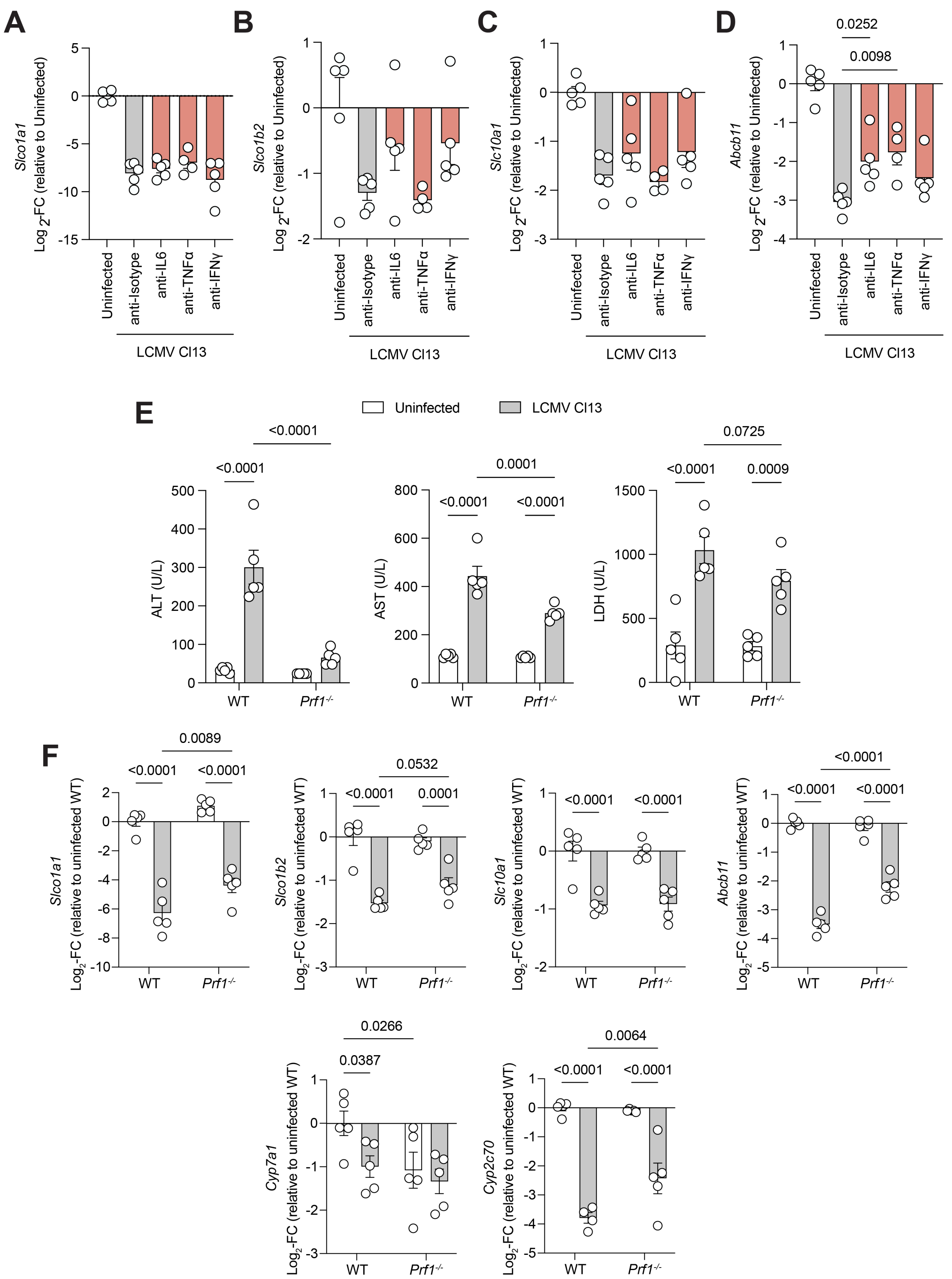

Supplementary Figure 4: Hepatic BA metabolism gene expression upon cytokine blockade and in *Prf1*-deficient mice. (A-D) Expression of hepatic BA receptors in response to LCMV Cl13 and antibody-mediated cytokine blockade at day 8 post-infection. (E) Circulating levels of tissue damage marker ALT, AST and LDH at day 8 post-LCMV Cl13

infection in wild-type and Perforin-deficient (*Prf1*$^{-/-}$) mice. (F) Hepatic BA gene expression at day 8 post-infection in wild-type and *Prf1*$^{-/-}$ mice.

Representative data from three independent experiments (n = 4-5 mice/group) and analyzed using One-Way ANOVA with Dunnett's multiple comparisons test (A-D). Data representative for two independent experiments (n = 5 mice/group) and analyzed using Two-Way ANOVA with uncorrected Fisher's LSD (E-F).

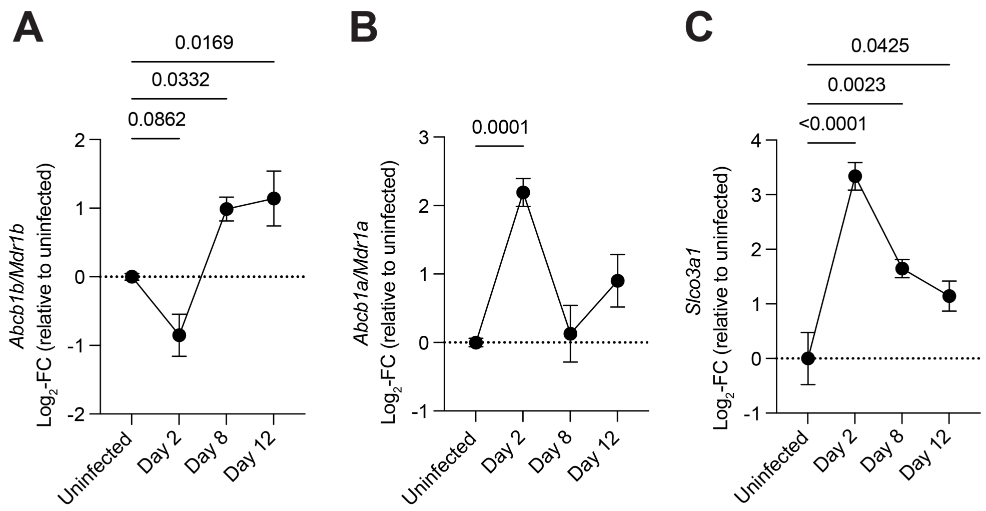

Supplementary Figure 5: Increased *Mdr1* and *Slco3a1* expression on total splenic $CD8^+$ T cells. (A) Expression of *Abcb1a/Mdr1a* in total $CD8^+$ T cells isolated at different time points post-LCMV Cl13 infection. (B) Expression of *Abcb1b/Mdr1b* in total $CD8^+$ T cells isolated at different time points post-LCMV Cl13 infection. (C) Expression of *Slco3a1* in total $CD8^+$ T cells isolated at different time points post-LCMV Cl13 infection.

Data pooled from two independent experiments (n = 6 mice/group) and analyzed using One-Way ANOVA with Dunnett's multiple test correction (A-C).

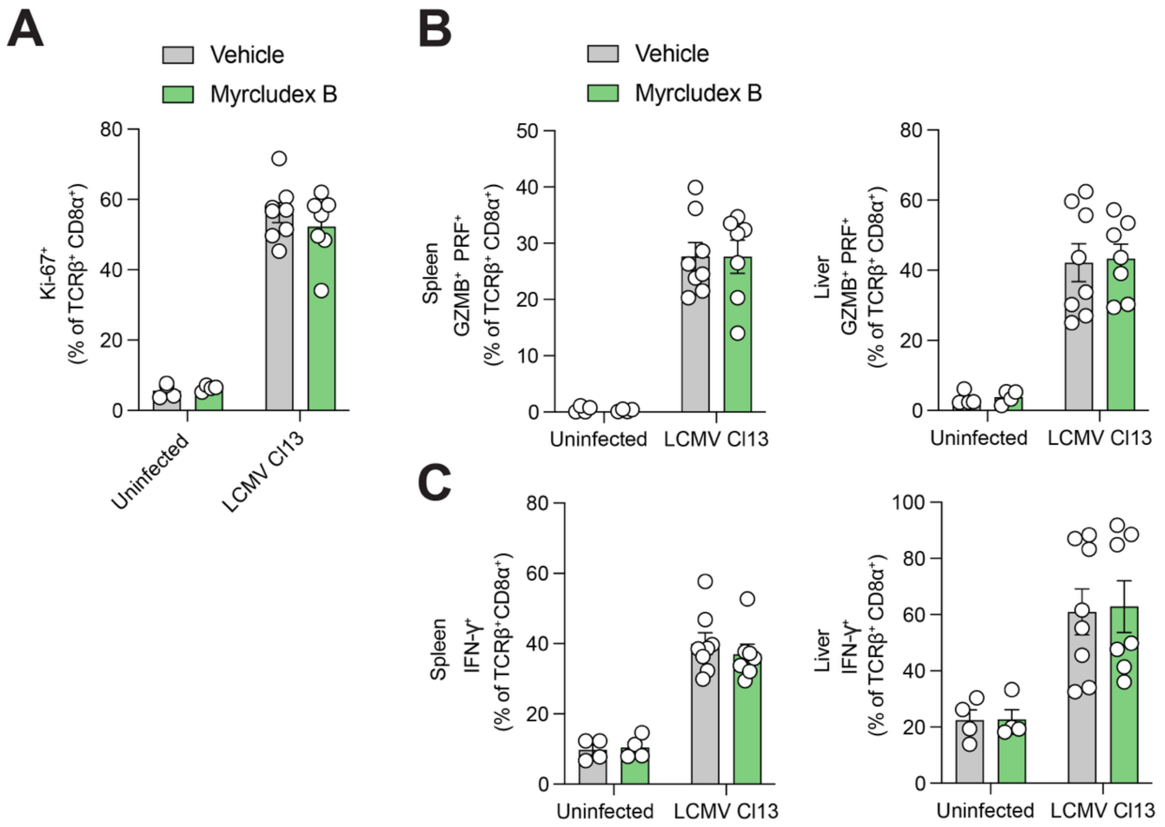

Supplementary Figure 6: Unaltered CD8$^+$ proliferation and function upon Myrcludex B treatment. (A) Frequency of proliferative CD8$^+$ T cells, marked by Ki-67 expression, in spleen at day 8 post-infection. (B) Frequency of cytotoxic CD8$^+$ T cells expressing Granzyme B (GZMB) and Perforin (PRF) in spleen and liver at day 8 post-LCMV infection. (C) Frequency of IFN-γ-producing CD8$^+$ T cells in spleen and liver at day 8 post-LCMV infection.

Data pooled from two independent experiment (n = 4-8 mice/group) and analyzed using Two-Way ANOVA with uncorrected Fisher's LSD (A-C).

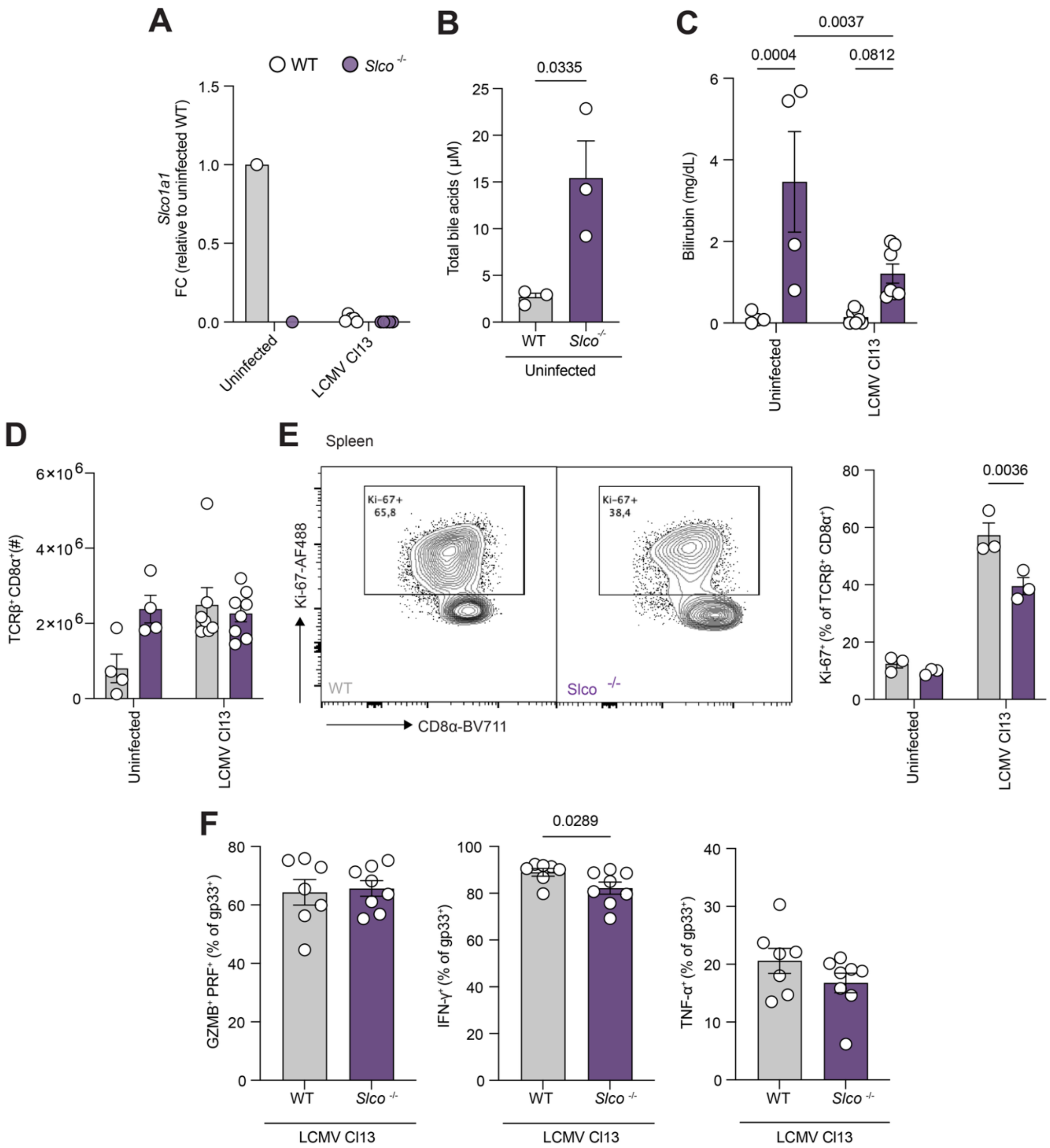

Supplementary Figure 7: Loss of BA transporter increased total BA and bilirubin levels, and is associated with changes in CD8^{+} T cell numbers and proliferation.

(A-C) Assessment of the known effects of *Slco*-deficiency in C57BL6/J background. (A) Expression of *Slco1a1* in the liver in *Slco*$^{-/-}$ and littermate controls upon infection with LCMV Cl13. Data representative for two independent experiments (n = 1-5 mice/group). (B) Total serum BA levels in uninfected *Slco*$^{-/-}$ and littermate controls. Data representative for two independent experiments (n = 3 mice/group) and analyzed with unpaired Student's t-test. (C)

Serum bilirubin levels in *Slco*$^{-/-}$ and littermate controls upon infection with LCMV Cl13. Data pooled from two independent experiments (n = 4-7 mice/group) and analyzed using Two-Way ANOVA with uncorrected Fischer’s LSD. (D-E) Total number of CD8^{+} T cells in the spleen (D) of *Slco*$^{-/-}$ animals and littermate controls. Data pooled from two independent experiments (n = 4-8 mice/group) and analyzed using Two-Way ANOVA with Šídák's multiple comparisons test. (E) Representative flow cytometry plot for Ki-67-expression of CD8^{+} T cell in the spleen 8 days post LCMV Cl13 infection. Data representative for one independent experiment (n = 3 mice/group) and analyzed using Two-Way ANOVA with Šídák's multiple comparisons test. (F) Cytokine and cytolytic enzyme expression of CD8^{+} T cells in spleen 8 days post LCMV Cl13 infection. Data pooled from two independent experiments (n = 4-8 mice/group) and analyzed using Two-Way ANOVA with Šídák's multiple comparisons test.

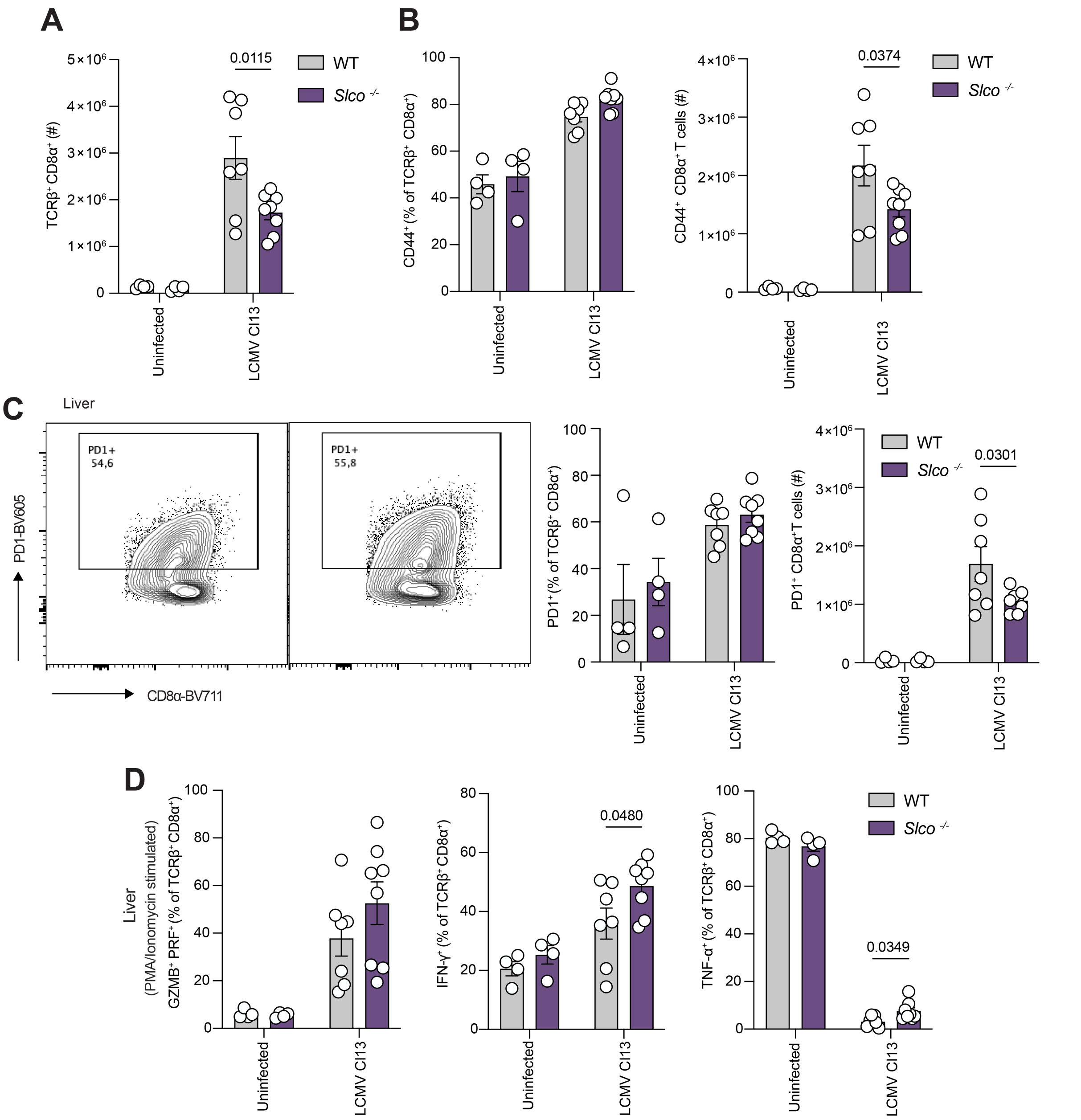

Supplementary Figure 8: CD8+ T cell infiltrating in the liver of *Slco*$^{-/-}$ mice is reduced, but virus-specific CD8+ T cells are functional. (A) Total number of CD8+ T cells in the liver of *Slco*$^{-/-}$ animals and littermate controls. (B,C) Frequency of (B) CD44- and (C) PD1-expressing CD8+ T cells in the liver at day 8 post-LCMV Cl13 infection. (D) Assessment of cytokine production in liver CD8+ T cells, isolated at day 8 post LCMV Cl13 infection, upon restimulation with PMA and ionomycin for 4h.

Data pooled from two independent experiments (n = 4-8 mice/group) and analyzed using Two-Way ANOVA with Šídák's multiple comparisons test.

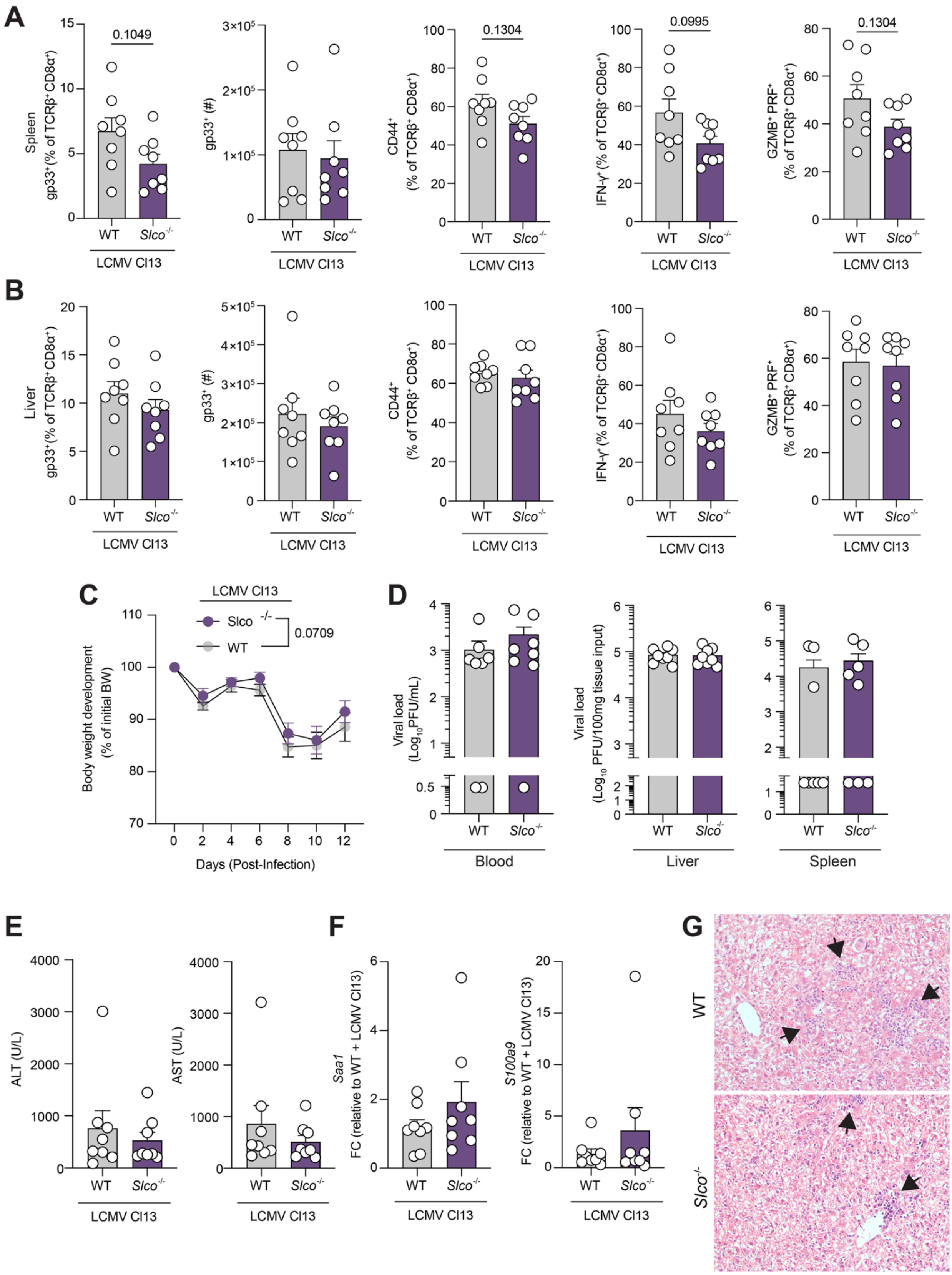

Supplementary Figure 9: *Slco*$^{-/-}$ exhibit similar immunopathology at day 12 post-LCMV Cl13 infection compared to littermate controls. (A) Total numbers and frequencies of virus-

specific $CD8^+$ and frequency of $CD44^+$, IFN- γ^+ and $GZMB^+$ PRF^+ $CD8^+$ T cells in the spleen. (B) Total numbers and frequencies of virus-specific $CD8^+$ and frequency of $CD44^+$, IFN-γ^+ and $GZMB^+$ PRF^+ $CD8^+$ T cells in the spleen. (C) Body weight development upon LCMV Cl13 infection. (D) Viral loads in indicated organs at 12 days post-infection. (E) Serum ALT and AST levels upon LCMV Cl13 infection in *Slco*$^{-/-}$ and littermate controls. (F) Hepatic expression of acute phase proteins at 12 days post-LMCV Cl13 infection. (G) Representative H&E images indicating liver pathology (Magnification 20X). Arrows indicate immune cell infiltrations. Hepatocyte ballooning was visible in both conditions.

Data pooled from two independent experiments (n=8 mice/group) and analyzed using Mann-Whitney test (A,B, D-F) and Two-Way ANOVA (C).

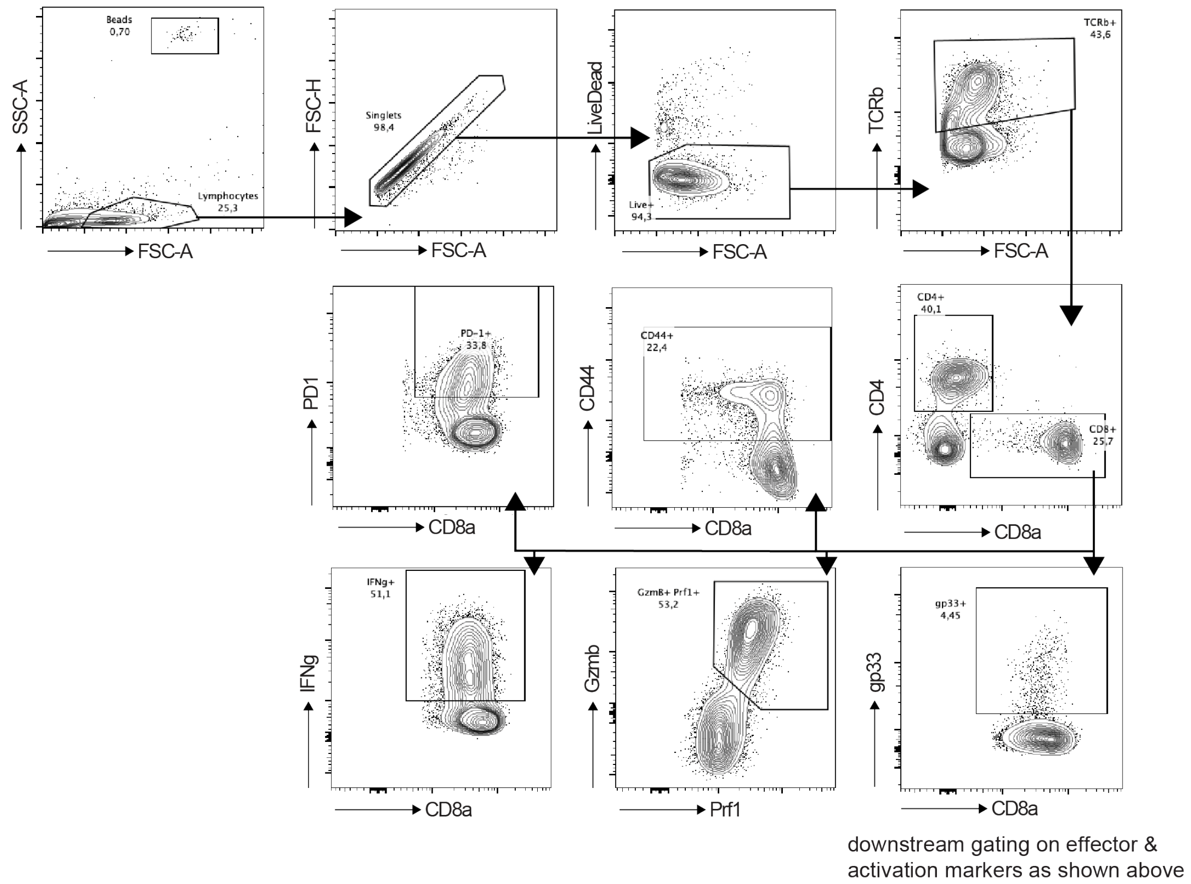

Supplementary Figure 10: Representative FACS gating strategy for CD8^{+} T cell characterisation. Lymphocytes were gated for single cells and viability. Then we used TCRβ^{+} cells to gate onto CD4^{+} and CD8a^{+} T cell subsets. From TCRβ^{+} CD8α^{+} T cells, we gated for cells specific for the viral epitope $GP_{33\text{-}41}$ using tetramers loaded with the peptide. Subsequently we gated for effector markers (CD44^{+}; PD1^{+}), cytokines (IFN-γ^{+}) and cytolytic markers (PRF^{+}; GZMB^{+}).
